# The SUMO Conjugase Ubc9 Protects Dopaminergic Cells from Cytotoxicity and Enhances the Stability of α-Synuclein in Parkinson’s Disease Models

**DOI:** 10.1523/ENEURO.0134-20.2020

**Published:** 2020-09-22

**Authors:** Dinesh Kumar Verma, Anurupa Ghosh, Lindsey Ruggiero, Etienne Cartier, Eric Janezic, Dionne Williams, Eui-Gil Jung, Michael Moore, Jong Bok Seo, Yong-Hwan Kim

**Affiliations:** 1 Department of Biological Sciences/Neuroscience program, Delaware State University, Dover, DE 19901; 2 Imaging Core, Delaware State University, Dover, DE 19901; 3 Seoul Center, Korea Basic Science Institute, Seoul 02841, Republic of Korea

**Keywords:** α-synuclein, degradation, lysosome, proteostasis, SUMOylation, Ubc9

## Abstract

Small ubiquitin-like modifier (SUMO) is a widespread regulatory mechanism of post-translational modification (PTM) that induces rapid and reversible changes in protein function and stability. Using SUMO conjugase Ubc9-overexpressing or knock-down cells in Parkinson’s disease (PD) models, we demonstrate that SUMOylation protects dopaminergic cells against MPP+ or preformed fibrils (PFFs) of α-synuclein (α-syn)-induced toxicities in cell viability and cytotoxicity assays. In the mechanism of protection, Ubc9 overexpression significantly suppressed the MPP+ or PFF-induced reactive oxygen species (ROS) generation, while Ubc9-RNAi enhanced the toxicity-induced ROS production. Further, PFF-mediated protein aggregation was exacerbated by Ubc9-RNAi in thioflavin T staining, compared with NC1 controls. In cycloheximide (Chx)-based protein stability assays, higher protein level of α-syn was identified in Ubc9-enhanced green fluorescent protein (EGFP) than in EGFP cells. Since there was no difference in endogenous mRNA levels of α-syn between Ubc9 and EGFP cells in quantitative real-time PCR (qRT-PCR), we assessed the mechanisms of SUMO-mediated delayed α-syn degradation via MG132, proteasomal inhibitor, and PMA, lysosomal degradation inducer. Ubc9-mediated SUMOylated α-syn avoided PMA-induced lysosomal degradation because of its high solubility. Our results suggest that Ubc9 enhances the levels of SUMO1 and ubiquitin on α-syn and interrupts SUMO1 removal from α-syn. In immunohistochemistry, dopaminergic axon tips in the striatum and cell bodies in the substantia nigra from Ubc9-overexpressing transgenic mice were protected from MPTP toxicities compared with wild-type (WT) siblings. Our results support that SUMOylation can be a regulatory target to protect dopaminergic neurons from oxidative stress and protein aggregation, with the implication that high levels of SUMOylation in dopaminergic neurons can prevent the pathologic progression of PD.

## Significance Statement

We tested whether SUMOylation enhances the solubility of aggregation-prone proteins such as α-synuclein (α-syn) to prevent protein aggregation induced by oxidative stress and/or preformed fibrils (PFFs) of α-syn. Here, we demonstrate that high levels of SUMOylation mediated by Ubc9 overexpression protect dopaminergic cells from MPTP-induced (MPP+) or PFF-induced toxicities. The protective effects are derived from the inhibition of reactive oxygen species (ROS) generation and protein aggregation. Interestingly, SUMOylated α-syn avoided lysosomal degradation, which was not detrimental. Ubiquitin binding to lysine residues may not compete with small ubiquitin-like modifier (SUMO) binding to determine the protein half-life of α-syn. Our findings strongly suggest that the regulation of SUMO conjugation to α-syn can be a novel therapeutic target to prevent the formation of Lewy bodies and ROS generation.

## Introduction

The formation of intraneuronal inclusions called Lewy bodies, in which α-synuclein (α-syn) is their major protein component, is a hallmark of Parkinson’s disease (PD; Spillantini et al., 1997). Although the roles of α-syn in neurons are not well characterized, it is known to be involved in many vital cellular functions including modulating vesicle trafficking (Pranke et al., 2011; Scott and Roy, 2012), regulating dopamine biosynthesis (Peng et al., 2005), preventing oxidation of unsaturated lipids in vesicles (Zhu et al., 2006), acting as a molecular chaperon (Chandra et al., 2005), maintaining the SNARE complex (Burré et al., 2010; Choi et al., 2013), as well as acting as a neuroprotective protein in dopaminergic cells (Jin et al., 2011). Pathologic mechanisms associated with PD have been reported that impaired proteasomal function or mutations of α-syn (e.g., A53T) can enhance its aggregation in the striatum and substantia nigra pars compacta (SNpc; Spillantini et al., 1997; Siderowf and Stern, 2003). Among the causes of α-syn aggregation, oxidative stress has often been reported to lead to the formation of insoluble cytotoxic α-syn species (Vijayakumaran et al., 2015). Therefore, it is crucial to understand the regulatory mechanisms of α-syn related to its solubility and/or its aggregation post-translationally for elucidating PD neuropathology. α-Syn is known to undergo numerous post-translational modifications (PTMs). For example, its phosphorylation, ubiquitination, nitration, acetylation, and SUMOylation have been reported to play numerous roles in modulating α-syn aggregation and toxicity (Giasson et al., 2000; Shimura et al., 2001; Fujiwara et al., 2002; Kim et al., 2011b; Popova et al., 2015; Kleinknecht et al., 2016). Ubiquitinated α-syn is typically targeted to proteasomal or lysosomal degradation (Pan et al., 2008). Thus, impaired proteasome function enhances the aggregation of α-syn, and ubiquitinated proteins including α-syn have been shown to aggregate in SNpc neurons (Spillantini et al., 1997; Eriksen et al., 2003).

SUMOylation is a reversible covalent modification that conjugates a small ubiquitin-like modifier (SUMO) protein to lysine in SUMO consensus domain in three separate steps such as SUMO activating enzyme (SAE1), Ubc9 and SUMO-E3 ligase (Wilkinson and Henley, 2010). Ubc9 is an E2-SUMO conjugase that covalently attaches a SUMO protein to α-syn at lysine residues if they are within a defined specific consensus site (ψ-K-x-D/E), where ψ corresponds to a large hydrophobic residue, K stands for lysine, x can be any amino acid, and D/E are glutamate or aspartate (Rodriguez et al., 2001; Sampson et al., 2001). SUMO tagging is known to enhance the solubility of conjugated proteins and may play a role in regulating the solubility of aggregation-prone proteins (Marblestone et al., 2006; Krumova et al., 2011). However, SUMOylation can be a major mechanism that counteracts ubiquitination by different E3 ubiquitin ligases (parkin) and regulates α-syn degradation (Rott et al., 2017). SUMOylated proteins have also been detected in the halo of Lewy bodies, co-localizing with α-syn in brains of patients with either PD or dementia with Lewy bodies (DLB; Kim et al., 2011b). Thus, SUMOylation and ubiquitination of α-syn in Lewy bodies may be regulatory mechanisms, modulating its degradation reciprocally in PD pathologic aggregation. Interestingly, UBC9 overexpression was reported to increase the solubility of α-syn and prevent methamphetamine (METH)-induced protein aggregation. Further, non-SUMO α-syn mutants enhance their aggregation by impairing proteasomal and lysosomal degradation (Zhu et al., 2018). Since SUMOylation of target proteins has been posited as an important factor in the pathogenesis or progression of PD, we assessed the effect of SUMOylation on α-syn for preventing its protein aggregation. The question of whether SUMOylation of α-syn is a good or bad modulation remains unresolved (Eckermann, 2013). Hence, the causal relationship between SUMOylation and α-syn aggregation/degradation and the relevance to α-syn toxicity need to be investigated.

A previous study showed that Ubc9 overexpression increases the protein level of dopamine transporter (DAT) and further enhances the functional activity of DAT with decreasing ubiquitination and degradation of DAT (Cartier et al., 2019). Here, we demonstrate a comprehensive role for SUMOylated α-syn in protein aggregation/degradation *in vitro*. Our results support that Ubc9-mediated SUMOylation is neuroprotective against MPTP (MPP+) or preformed fibrils (PFFs) of α-syn-induced toxicities *in vivo* and *in vitro* (Volpicelli-Daley et al., 2011). Intriguingly, we found that SUMOylated α-syn was refractory to normal lysosomal degradation, however, this aberrant degradation was not detrimental to α-syn-mediated protein aggregation because of the increased solubility (Krumova et al., 2011). Our study strongly suggests that SUMOylation is a novel mechanism that plays a crucial role in regulating α-syn solubility and degradation in dopaminergic cells, and it can be a therapeutic target for PD.

## Material and Methods

### Animals

Ubc9-overexpressing transgenic (Ubc9-Tg) C57BL/6 background mice (30–45 g) were received as a gift from John Hallenbeck at NINDS (Lee et al., 2011, 2014). All animal protocols were conducted in accordance with the United States Public Health Service Guide for the Care and Use of Laboratory Animals; all procedures were approved by the Institutional Animal Care and Use Committee (IACUC). Four or five animals per polyacrylic cage were housed with access to food and water *ad libitum* and were maintained in standard housing conditions, i.e., at room temperature (RT) 24 ± 1°C and humidity 60–65% with 12/12 h light/dark cycle.

### Animal groups and treatment

All the Ubc9-Tg mice (males and females) are hemizygous and their wild-type (WT) siblings were used as controls. When mice were aged up to 11–12 months old, 25 μg/g body weight of MPTP (dissolved in 0.9% saline, Sigma) was injected once a day for seven consecutive days (Lazzara et al., 2015). Since MPTP toxicity is strain and age sensitive, the chronic injection to the same age mice was performed at the same time for minimizing the variation of MPTP toxicity (*n* = 10 per group). In the vehicle group, the same volume of 0.9% saline was injected (*n* = 10). Seven days after the last injection, mice were deeply anesthetized in an isoflurane chamber and intra-cardiac perfusion was done by using ice-cold 0.9% saline, followed by 150 mm NaCl/70% ethanol. After decapitation, whole brains were isolated. Half of each brain was isolated by brain-region, e.g., the striatum and brain stem, and stored at −80°C for molecular/biochemical analyses including reactive oxygen species (ROS) measurement; the other half brain was postfixed in 4% paraformaldehyde (PFA) for immunohistochemical analysis.

### Cell lines with plasmids or RNAi treatment

N27 rat dopaminergic parental cell line (SCC048, EMD Millipore) was used to generate enhanced green fluorescent protein (EGFP) or Ubc9-EGFP-overexpressing (Ubc9-OE) stable cell lines by transfection. Neomycin resistant plasmids containing EGFP (based on pEGFP vectors, Clontech; now Takara Bio USA) or Ubc9-EGFP (a gift from Jo Morris, King’s College, London) were transfected to N27 cells using Lipofectamine 3000 (Thermo) as per the manufacturer’s instructions. All cell lines, including EGFP, Ubc9-OE, and rat dopaminergic parental cell line (N27p), were cultured in RPMI 1640 (Invitrogen) cell culture media along with 10% FBS (Atlanta Bio) and 1% penicillin-streptomycin (Invitrogen) in a 5% CO_2_ incubator at 37°C. Forty-eight hours after transfection, G418 (Geneticin, Invitrogen) was added as a selection marker at the concentration of 500–1000 μg/ml for two weeks. Clusters of green fluorescent-positive (EGFP) cells were isolated and plated into 60 mm dishes. G418 (500 μg/ml) was added for another two weeks and then lowered to 200 μg/ml as previously reported (Cartier et al., 2019). To knockdown Ubc9 expression, N27p cells were transfected with dicer substrate siRNAs (RNAi-N013050.12.1 5′-UCCGUACAGUUACUAGUA-3′ 3′-UCAAUGAUCAUCGGACCC-5′, RNAi-N013050.12.3 5′-GUACGAUGAACGUGAUGA-3′ 3′-CUVGGACUACUUGACCCU-5′ or negative control NC1 purchased from IDT using Lipofectamine 3000 (Thermo), according to the manufacturer’s directions. After the exposure overnight, NC1 or Ubc9-RNAi-treated cells were exposed to MPP+ or PFF for 24 h, followed by cellular measurements including cell viability, cytotoxicity, ROS, and thioflavin T staining, etc.

### Cell culture and treatment

MPP+ (1-methyl-4-phenylpyridinium, Sigma) treatment was applied at concentrations of 160, 320, 640, and 1280 μm for 24 h. The PFFs of α-syn was purchased from StressMarq Biosciences (SPR-324C) and its treatment was tested at 1, 2, 3, 4, and 5 μg/ml for 24 h. Cells were also treated with cycloheximide (Chx; Sigma), protein synthesis inhibitor (100, 150, and 200 μg/ml) for 24 h and PMA, PKC-mediated lysosomal inducer (2, 4, 6, 8, and 10 μm) for 2 h. Additional experiments were performed using 640 μm MPP+, 150 μg/ml of Chx, and 5 μm PMA for 24-h exposure. There were also co-treatments of chloroquine (lysosomal inhibitor; 10 μm) and MG132 (proteasomal inhibitor; 10 μm), also known as Z-Leu-Leu-Leu-al for 24 h (Cartier et al., 2019).

### Cell viability assay

The conversion of MTT dye to soluble formazan is caused by mitochondrial dehydrogenase enzymes in live cells. The formazan is soluble in DMSO and produces purple/blue color that indicates the level of cell viability. The cell viability in the vehicle group of EGFP and Ubc9 cells was calculated as 100% of control. Cell viability was estimated by using the dye 3-(4,5-dimethylthiazol-2-yl)−2,5-diphenyltetrazolium bromide (MTT), following the protocol described by Janezic et al. (2016). A total of 5000 cells were seeded in 96-well plates. After 1 h, cells were treated with different concentrations of MPP+ or PFF (1 μg/ml) for 24 h. MTT (5 μm) dye was added into the culture media and incubated at 37°C for 2 h. The culture medium was removed, and 200 μl of DMSO was added to each well. The purple-colored formazan was dissolved in DMSO and color intensity was measured at 570 nm with a reference wavelength of 630 nm by using a spectrophotometer (SpectraMax M5^e^, Molecular devices). The measured optical density is directly correlated with cell viability.

### Cytotoxicity assay

By measuring the level of lactate dehydrogenase (LDH; a soluble cytosolic enzyme), released into the culture medium on cell lysis, the level of cytotoxicity was assessed. The cytotoxicity assay was performed by using Pierce LDH cytotoxicity assay kit (88954, Thermo), as per the manufacturer’s protocol. Briefly, after treatment with different concentrations of MPP+ or PFF for 24 h, the cell culture media were collected for LDH assay, and cells were further used for cell viability (MTT) assay. The protein concentration was measured by using Pierce Rapid Gold BCA protein assay kit (A5225, Thermo Fisher Scientific) as per the manufacturer’s protocol. For LDH assay, 50 μl of cell culture media per well was transferred into a new 96-well plate. Then, 50 μl of reaction mixture was added to each well, and a plate was incubated at RT for 30 min. After incubation, 50 μl of stop solution was added to each well. Absorbance changes were measured at 490 and 680 nm by a spectrophotometer (SpectraMax M5e, Molecular Devices). To determine LDH activity (%), 680-nm absorbance values were subtracted from the readings at 490-nm absorbance, and then the normalized values were calculated in the scale of vehicle-treated control.

### ROS level measurement

The level of ROS was assessed by using CellROX deep red reagent (C10422, Thermo Fisher Scientific) as the manufacturer’s protocol instructed. Briefly, after treatment of MPP+ or PFF, CellROX (5 μm) was added into the cell culture medium in duplicate and incubated at 37°C for 30 min. The cells were then washed with PBS and images were randomly captured using the EVOS FL Cell Imaging Systems (Invitrogen). After four to five independent experiments (total replicates: *n* = 8–10), the intensity of red color was analyzed by a blinded rater using ImageJ.

### Thioflavin T and α-syn staining

For double staining, up to 4.5 × 104 N27p cells were seeded per well on poly-D-lysine-coated glass coverslips in six-well plates. After transient transfection using Lipofectamine 3000 with Ubc9-RNAi or NC1 treatment overnight, cells were treated with wheat germ agglutinin/N-acetylglucosamine (WGA/GluNAc, Sigma) for PFF (1 μg/ml) penetration as described by Volpicelli-Daley et al. (2011). Briefly, cells were fixed with 4% PFA/PBS solution for 20 min and washed three times in PBS. Blocking and permeabilization was done using 2% bovine serum albumin (BSA) prepared in PBS-T (0.25% Triton X-100) at RT for 1 h. Then, cells were incubated with anti-α-syn primary antibodies (1:250, MABN1817, Sigma-Millipore) at RT for 2 h and washed in PBS three times. The cells were treated with Alexa Fluor 647-conjugated secondary antibodies (1:1000; Molecular probes, Thermo Scientific) at RT for 1 h and washed in PBS three times. For thioflavin T staining, we have followed the procedures from the recent publication (Polinski et al., 2018) with minor modifications. Cells were incubated with 20 μm thioflavin T (Sigma-Aldrich) solution at RT for 30 min and washed in PBS three times. After staining, cell images were randomly captured using fluorescence microscopy (EVOS, Invitrogen) using proper optical filters. As a negative control, the secondary antibody step or the thioflavin T step was omitted. After capturing random cell images, the relative fluorescent intensity of thioflavin T (green) or α-syn (red) was compared with control (WGA/NAcGlu, 100%) for assessing the effect of PFF or Ubc9-RNAi. All the experiments were independently run at least four times in duplicate (*n* ≥ 8 per group) and three to five images per well were captured for thioflavin T or α-syn. Thus, total images (*n* = 24–40) per group were analyzed by a blinded rater.

### Immunocytochemistry

N27 cells were seeded on poly-D-lysine-coated glass coverslips per well in 6-well plates. Cells were washed in ice-cold PBS without Ca^2+^ or Mg^2+^ (137 mm NaCl, 2.7 mm KCl, 4.3 mm Na_2_HPO_4_, and 1.47 mm KH_2_PO_4_) for 5 min three times, followed by fixation with 4% PFA at RT for 20 min and washed in PBS for 5 min three times. Fixed cells were permeabilized with 0.1% Triton X-100 and 0.5% BSA for 3 min, followed by blocking with 5% BSA at RT for 2 h. Then, cells were incubated with mouse anti-α-syn primary antibodies (1:500, MABN1817, Sigma-Millipore) in 1% BSA/PBS at 4°C overnight. On the following day, cells were washed in 0.1% PBS-T (Triton X-100) three times for 5 min each and incubated with secondary goat anti-mouse Alexa Fluor 594 (1:1000; Invitrogen, Thermo Fisher Scientific) at RT for 1 h. Cells were washed in PBS for 3 × 5 min, followed by a brief washing in ultrapure water. Coverslips were mounted on glass slides using ProLong Diamond antifade mounting medium (P36961, Thermo). Images were obtained using Zeiss 780 Multiphoton Confocal microscopy at the imaging core in our institution. All images were acquired with a Plan-Apochromat 40×/1.4 NA Oil DIC objective (Zeiss). Images were processed using Zeiss ZEN software and sampled at optimal pixel density in the X and Y with a line average of 4, and a zoom factor of 1.2. Z-stacks were set up with optimal z-sectioning for optimal overlap and processed into maximum intensity projections.

### Immunohistochemistry

A series of total coronal sections throughout the striatum and substantia nigra were sectioned in 16-μm thickness and collected in total four sets for mounting onto positively charged slides (Midwest Sci). For rehydration, slides were immersed into xylene for 5 min twice, followed by washing in serially diluted ethanol (100% for 5 min twice; 95% for 5 min; 70% for 5 min; 50% for 5 min) and rinsed with deionized water. Slides were washed in PBS for 5 min twice. Antigen unmasking was done by using Tris-EDTA Buffer (10 mm Tris-base, 1 mm EDTA solution, and 0.05% Tween 20; pH 9.0) at RT for 15 min and washed in PBS for 5 min three times. After blocking in 2% BSA at RT for 30 min, slides were incubated with anti-tyrosine hydroxylase (TH) antibody (1:1000; AB152, EMD-Millipore) at 4°C overnight and washed in PBS-T for 5 min three times. Then slides were incubated with Alexa Fluor 647-conjugated goat secondary anti-mouse antibody (1:1000; Invitrogen, Thermo Fisher Scientific) at RT for 1 h and washed in PBS-T for 3 × 5 min. As a negative control, the primary antibody step was omitted. Coverslips were mounted on slides using ProLong Diamond antifade mounting medium (Thermo). Images were acquired using LSM 510 confocal microscope with Z-stacks (0.5 μm) which were set up with optimal z-sectioning for optimal overlap at the internal imaging core. All images were acquired with a Plan-Apochromat 40×/1.4 NA oil DIC objective. Images were processed into maximum intensity projections using Zeiss ZEN software. Every fourth section was analyzed for measuring the intensity of TH staining in the striatum and for counting TH+ neurons in the substantia nigra compacta (SNc) in a blinded manner.

### α-Syn quantitative mRNA measurement

Using quantitative real-time PCR (qRT-PCR; LightCyler 480II, Roche), the level of α-syn mRNA was determined with β-actin as a housekeeping gene. An equal number of N27 cells overexpressing either Ubc9 or EGFP was collected for RNA extraction using the TRIzol Reagent (Life Technologies). The experimental procedures were repeated, according to the recent report (Cartier et al., 2019) except for the primer information below: the parameters for the reactions were: 95°C for 10 min, followed by 50 cycles of 95°C for 30 s, 60°C for 10 s, and 72°C for 10 s. The fluorescence was recorded during the 72°C step to determine the Crossing point (Cp) value. The primers were chosen based on Primer3Plus software and synthesized by the Integrated DNA Technologies (IDT). The primers are: α-syn forward 5′-GCAGTGAGGCTTATGAAATGC-3′; α-synuclein reverse 5′-AGGCTTCAGGCTCATAGTCTTG-3′; β-actin forward 5′-AGCCATGTACGTAGCCATCC-3′; β-actin reverse 5′-CTCTCAGCTGTGGTGGTGAA-3′. Each trial was performed in triplicate, and three trials were collected for total of nine independent samples for each gene.

### Chx protein chase

EGFP or Ubc9 cells were equally plated (1 × 105 cells/plate) on 60-mm dishes and incubated with different concentrations of Chx (100, 150, and 200 μg/ml) in the culture media overnight. Additional experiments were performed using 150 μg/ml Chx at different time points (T = 0, 6, 12, 18, or 24 h). Cells were lysed using 250-μl RIPA buffer [50 mm Tris, 100 nm NaCl, 1% NP-40, 1 mm sodium fluoride (NaF), 2 mm sodium orthovandate (Na_3_VO_4_), 10 mm NEM, 1 mm PMSF, and 1% protease phosphatase inhibitors] and sonicated briefly on ice for solubilization (3 s on and 10 s off for five cycles). Treatment or vehicle was added to the media at T = 0 h and remained for the rest of chase studies, which was terminated as described previously (Cartier et al., 2019). Cell lysate was centrifuged at 15,000 × *g* for 10 min, and supernatant was collected and separated by 4–20% SDS-PAGE gels (GenScript). α-Syn was detected by immunoblotting as described below in Immunoblot analyses.

### Immunoprecipitation (IP)

After treatment, cells were washed in 1× PBS twice and lysed in 250 μl of RIPA buffer with incubation for 15–20 min. After brief sonication, cell extracts were centrifuged at 15,000 × *g* for 10 min. The supernatant was transferred into a fresh vial and protein concentration was measured by using Pierce Rapid Gold BCA Protein Assay kit (A53225, Thermo Fisher Scientific). An equal amount of protein (500 μg) was taken for IP assay, and samples were precleared with A/G beads (Santa Cruz Biotech) at 4°C for 1 h. To remove A/G beads, brief centrifugation was followed by collecting the supernatant for transferring to a fresh vial. Then, equal amounts of anti-α-syn antibody (MABN1817, EMD-Millipore) were added to each sample and incubated at 4°C overnight. On the following day, equal amounts of A/G beads were added to each sample. After 2 h of incubation, samples were collected and mixed with non-reducing sample loading buffer (39001, Thermo Fisher Scientific). All Immunoprecipitated samples were separated by 4–20% SDS-PAGE gels (GenScript) along with 5% total lysate as input. SUMO1, ubiquitin and α-syn proteins were detected by immunoblotting as described below in Immunoblot analyses.

### Immunoblot analyses

Protein samples were loaded on precast polyacrylamide gel (SurePAGE Bis-Tris, 4–20%, 12 wells; GenScript) and transferred to PVDF membrane (Immobilon-P, EMD-Millipore) using Bio-Rad transfer apparatus. Membranes were incubated with anti-α-syn antibody (1:2000; MABN1817, EMD-Millipore), anti-α-syn antibody for IP samples (1:1000; 610787, BD Biosciences), anti-SUMO1 (1:1000, sc-5308, Santa Cruz Biotech), or anti-ubiquitin (1:1000, sc-8017, Santa Cruz Biotech) at 4°C overnight. Equal loading was determined by stripping and re-probing against glyceraldehyde-3-phosphate dehydrogenase (GAPDH; 1:10,000; AM4300, Thermo Fisher Scientific). After washing, membranes were incubated with secondary anti-mouse IgG antibodies conjugated with HRP (1:10,000) at RT for 2 h. The PageRuler-prestained protein ladder was used to estimate protein molecular weights on immunoblots (Thermo 26 616). Signals were developed in Immobilon Forte Western HRP substrate (EMD-Millipore) and detected under ChemiDoc iBright CL1000 (Invitrogen). The integrated density (intensity/area) of each band was measured and normalized by GAPDH (or α-syn in IPs) and/or total protein loading labeled by Reversible protein stain kit for PVDF membranes (Thermo, 24585) as a loading control. Immunoblot images were converted into eight-bit gray-scale images and avoided over-saturation. Equal areas corresponding to selected lanes were analyzed on each blot image using ImageJ software (NIH). Splicing was implemented only for clarity purposes and the adjustment was performed using the original larger image.

### Sample preparation and data analysis for mass spectrometry

Cell extracts from Ubc9-OE or EGFP N27 cells were IP’ed by α-syn antibodies (MABN1817, EMD Millipore) as described above and then run in 10% SDS-PAGE gel (GenScript). Three pieces per well were dissected based on the size of bands and each piece was separately rinsed once in 200 μl of ddH_2_O, twice in 200 μl of 25 mM ammonium bicarbonate in 50% (v/v) acetonitrile, followed by 100 μl of acetonitrile to dehydrate the isolated gel pieces, which were then lyophilized. The dry gel pieces were rehydrated in 150 μl of 25 mM ammonium bicarbonate containing 25 ng/μl trypsin (pH 8.0). After rehydration, additional 100 μl of 25 mm ammonium bicarbonate was added and the gel pieces were incubated at 37°C overnight. Digested samples were desalted using a Harvard apparatus micro-spin column containing C18 resin. The pooled extracts were reduced to dryness and reconstituted in 80% acetonitrile/0.1% formic acid for tims-TOF Pro mass spectrometry (Bruker), which was operated in PASEF mode using Compass Hystar 5.0.37.1. The experiments were repeated three times for quantification (*n* = 3).

Data files were uploaded to PEAKS X (Bioinformatics Solutions) for processing, *de novo* sequencing and database search. The sequences were searched based on the UniProt *Rattus norvegicus* database (37,157 entries) since N27 cells are rat originated. We searched with mass error tolerances of 20 ppm and 0.05 Da for parent and fragment, respectively. Trypsin enzyme specificity, acetylation (N-term), oxidation, and phosphorylation were adopted as variable modifications. Peptides were filtered at an 1% false discovery rate (*p* < 0.01) in the peptide spectrum match level, and protein filtering was disabled by setting proteins as −log10[P] score at two unique peptides with a requirement for significant peptides.

### Chemicals

Most chemicals in this study, such as phorbol 12-myristate 13-acetate (PMA; Sigma P1585), N-ethylmaleimide (NEM; Sigma E3876), Chx (Sigma C7698), chloroquine diphosphate (Sigma C6628), MG132 Z-Leu-Leu-Leu-al (Sigma C2211), lectin also known as WGA (Sigma L9640), N-acetylglucosamine (Sigma PHR1432), MPP+ (Sigma D048), thioflavin T (T3516), MPTP (M0896), NaF (201154), and Na_3_VO_4_ (567540) were purchased from EMD-Sigma. PFF of α-syn was obtained from StressMarq (SPR-324C) and PMSF (Roche 11359061001), BSA (Fisher bioreagent BP9703), and PBS (Invitrogen 10010031) were used as well. Protease inhibitor used was EDTA-free Halt protease inhibitor cocktail (Thermo Scientific 87785).

### Statistical analyses

**Figure 1.**
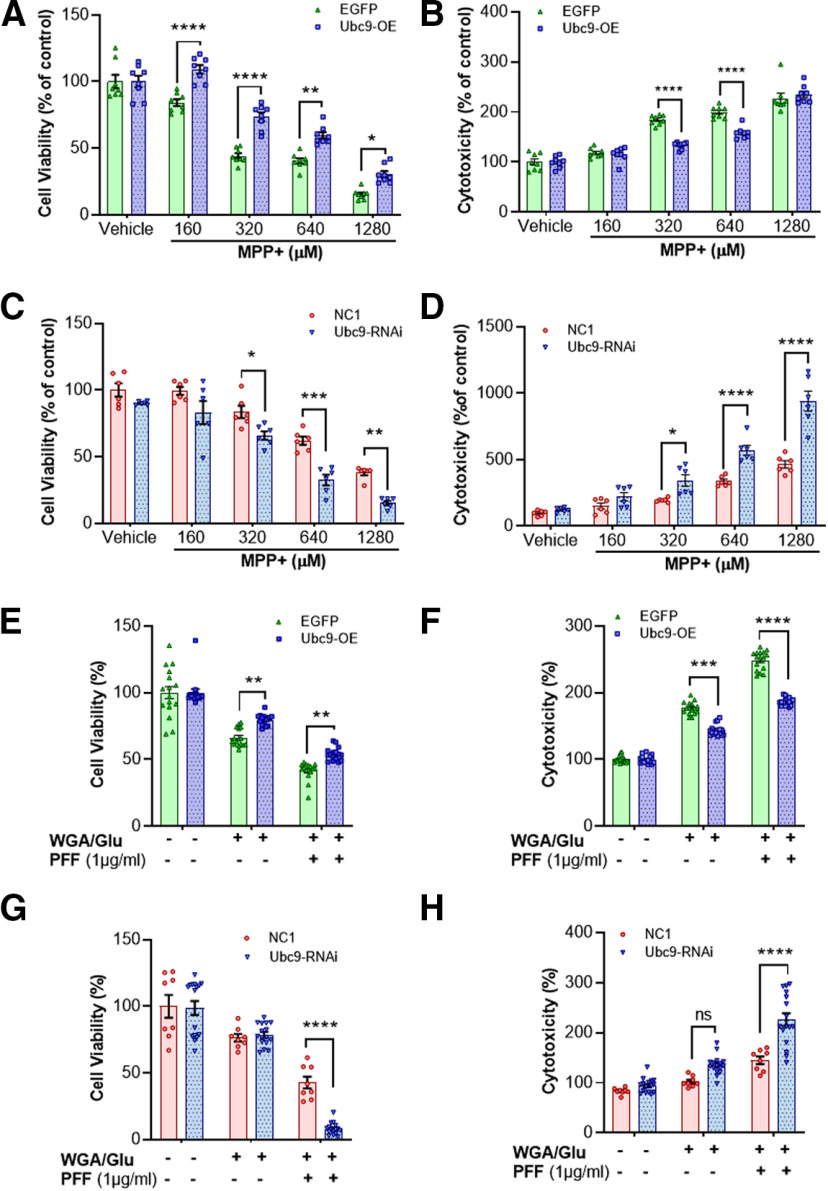
Ubc9 expression protects N27 cells from MPP+ or PFF-induced toxicity, enhancing cell viability and reducing cytotoxicity. ***A***, MPP+ exposure reduces the number of viable cells in EGFP cells in a dose-dependent manner, whereas Ubc9 overexpression protects N27 cells from MPP+ toxicity in MTT assay. ***B***, In LDH assay, Ubc9 overexpression reduces the toxic effect derived from MPP+ exposure. ***C***, In MTT assay, Ubc9 knock-down by RNAi exacerbates the cell viability induced by MPP+ exposure, compared with NC1 random cocktail control. ***D***, In LDH assay, Ubc9-RNAi significantly increases the cytotoxicity derived from MPP+ treatment. ***E***, In MTT assay, PFF treatment reduces cell viability in EGFP cells compared with WGA/GluNAc-treated control, while Ubc9 overexpression ameliorates the toxic effect from PFF. ***F***, In LDH assay, PFF-induced cytotoxicity was suppressed by Ubc9 overexpression. ***G***, In MTT assay, Ubc9-RNAi further exacerbates the cell viability induced by PFF treatment, compared with NC1 random cocktail control with WGA/GluNAc. ***H***, In LDH assay, Ubc9 knock-down substantially enhances the cytotoxicity derived from PFF treatment compared with NC1 control. All the treatments were exposed for 24 h, and each dot represents the number of experiments and each experiment was performed in triplicate. Statistical analysis was applied using two-way ANOVA, Tukey’s *post hoc* test. Scattered dot plots represent mean ± SEM; *p < 0.05, **p < 0.01, ***p < 0.001, ****p < 0.0001 (*n* = 6–15 per group).

**Figure 2.**
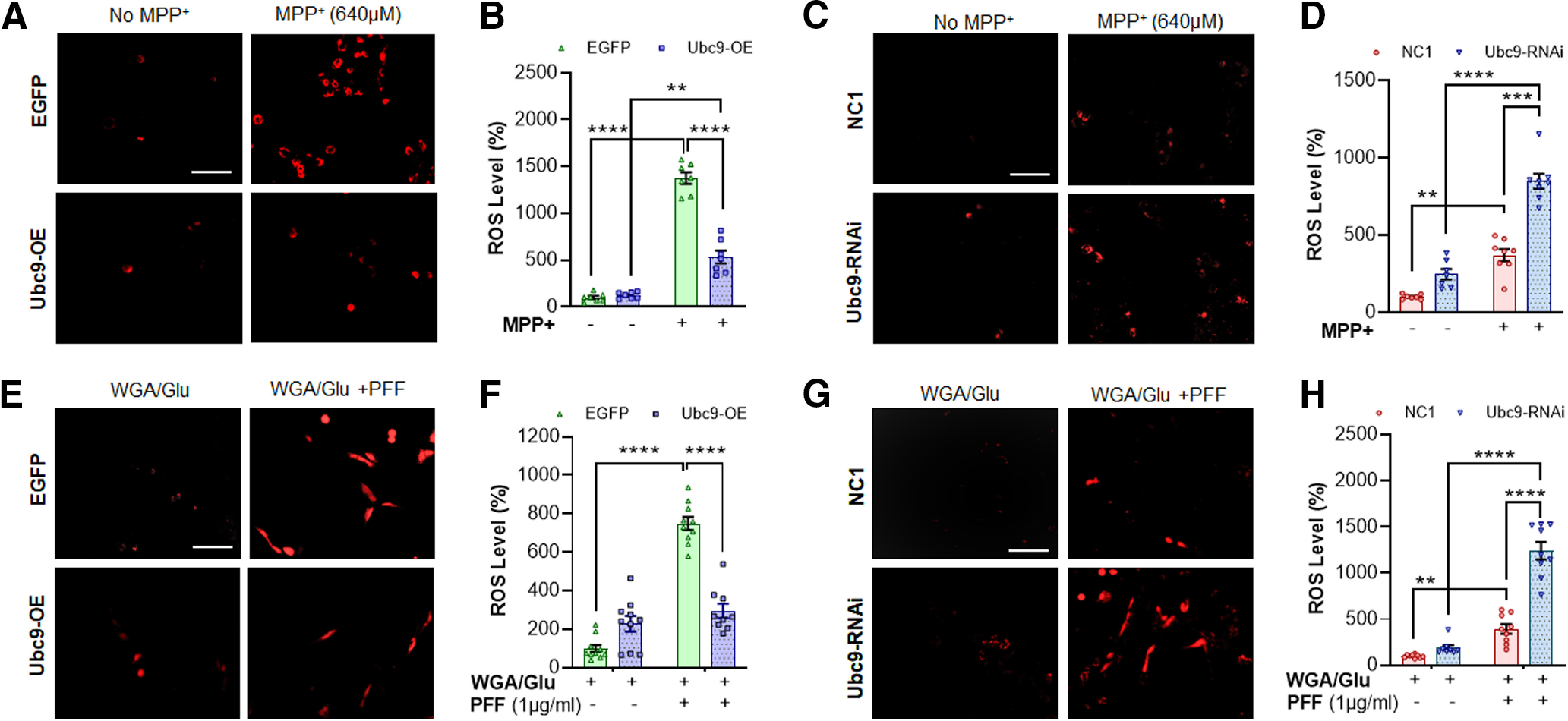
Ubc9 expression suppresses ROS generation triggered by MPP+ or PFF treatment. ***A***, Examples of ROS-labeled cells show that MPP+ (640 μm) for 24 h stimulates the generation of ROS in N27 EGFP cells, which was labeled with CellROX red fluorescent dye in dark field microscopy. ***B***, MPP+ triggers a striking increase in ROS generation, which was prevented by Ubc9 overexpression. ***C***, Examples of cell images showing that Ubc9-RNAi exacerbates the ROS production induced by MPP+ exposure. ***D***, The treatment of Ubc9-RNAi (100 pm) overnight enhances MPP+-induced ROS generation compared with NC1 random construct control. ***E***, Cell images show that PFF (1 μg/ml) exposure for 24 h stimulates ROS production (red label) in EGFP cells, which was not clearly detected with Ubc9 overexpression. ***F***, The ROS generation induced by PFF was almost completely suppressed by Ubc9 overexpression, compared with WGA/GluNAc control. ***G***, Cell images show that Ubc9-RNAi exacerbates the PFF-induced ROS production, compared with no treatment or WGA/GluNAc control. ***H***, Ubc9-RNAi significantly enhances ROS production induced by PFF treatment, compared with NC1 control. Integrated density of images was measured by ImageJ and presented in scattered dot plots. Statistical analysis was applied using two-way ANOVA followed by Tukey’s multiple comparisons *post hoc* test. All dot plots were displayed as individual values and mean differences are depicted as horizontal line (*n* = 7–10); SEM is indicated by the end of the vertical error bars; **p <0.01, ***p <0.001, ****p <0.0001 (*n* = 8–10 per group; *n* represents the average intensity of ROS in each well). Scale bar: 20 μm.

**Figure 3.**
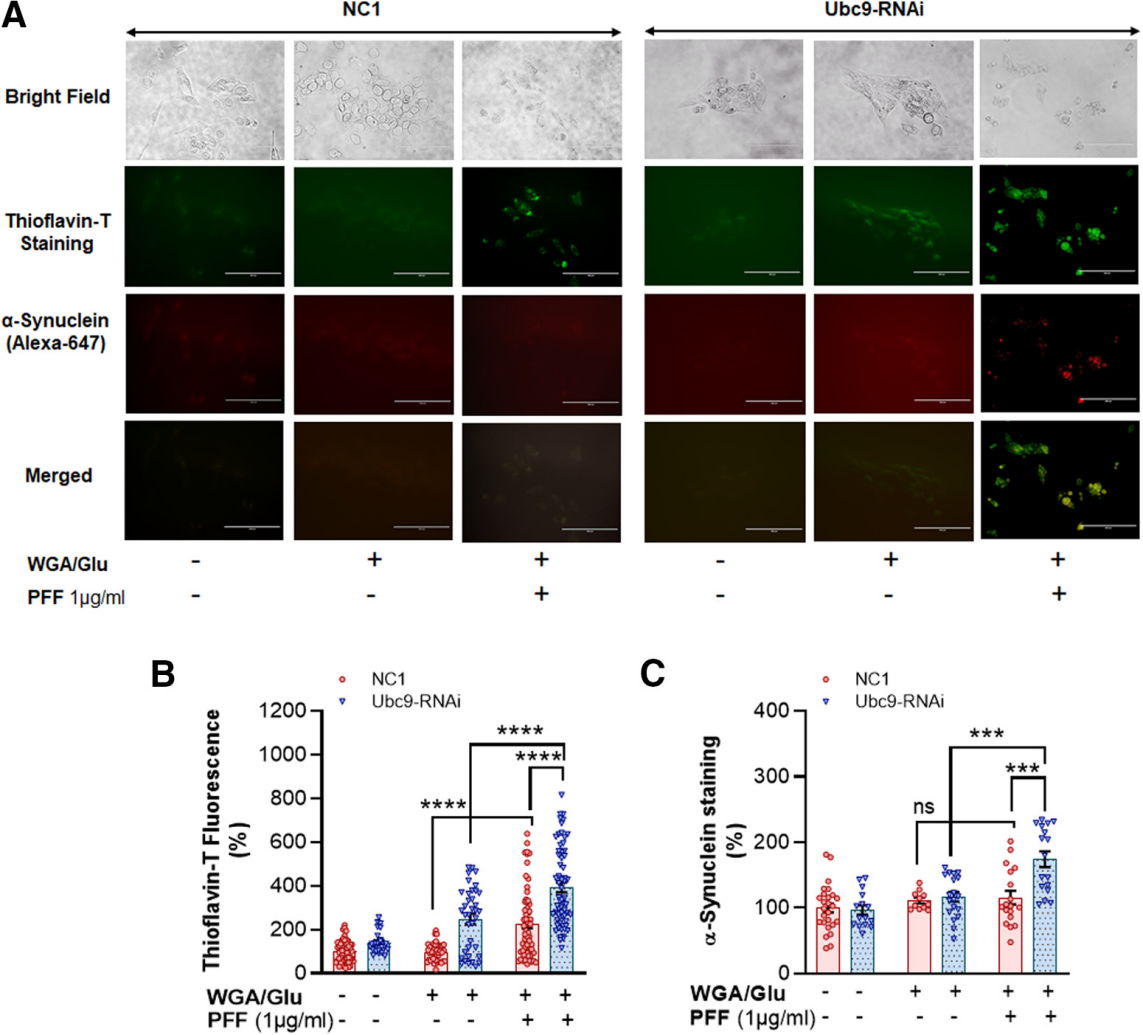
Ubc9 knock-down by RNAi exacerbates PFF-induced protein aggregation in thioflavin T staining. ***A***, Bright field images show the location of N27 cells (top row). Immunocytochemical images of thioflavin T staining in dark field show PFF-induced protein aggregation in green fluorescent label (second row). α-Syn staining (red) in immunocytochemistry was detected in thioflavin T -labeled protein aggregates (third row). The merged images (yellow, the bottom row) demonstrate that α-syn is co-localized with thioflavin T-stained protein aggregates. ***B***, PFF exposure to N27 cells for 24-h results in the accumulation of protein aggregation labeled in thioflavin T. Ubc9-RNAi further enhances PFF-induced protein aggregation, compared with NC1 random cocktail control with WGA/GluNAc. ***C***, PFF increases the level of α-syn in thioflavin T-positive aggregates with Ubc9-RNAi, and Ubc9 knock-down aggravates α-syn accumulation in the protein aggregates compared with NC1 control after PFF treatment, although PFF did not significantly increase the level of α-syn in the aggregates in NC1 control-treated cells. Integrated density of images was measured by ImageJ and presented in scattered dot plots. Statistical analysis was performed using two-way ANOVA followed by Tukey’s multiple comparisons *post hoc* test. All dot plots were displayed as individual values and mean differences are depicted as horizontal lines; SEM is indicated by the end of the vertical error bars; ***p < 0.001, ****p < 0.0001; ns: not significant (*n* = 24–40 per group). Scale bar: 100 μm.

**Figure 4.**
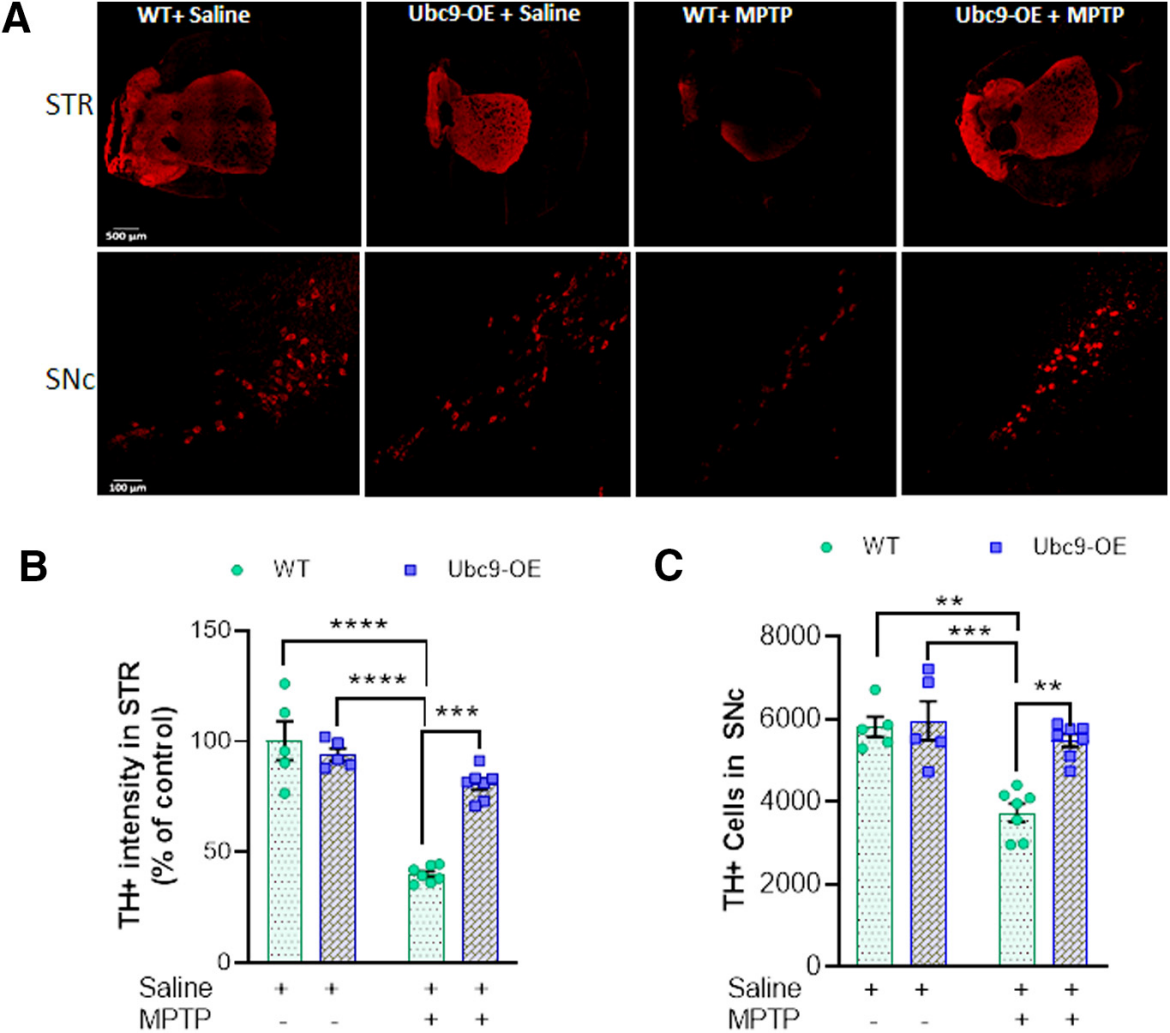
The overexpression of Ubc9 protects dopaminergic neurons from MPTP-induced toxicity in mouse brain. ***A***, Confocal images of the striatum (STR) and SNc region of C57Bl/6 mice after staining with anti-TH antibody (1:100) conjugated to Alexa Fluor 555 (1:250). ***B***, Quantitative analysis of TH+ nigrostriatal projection intensity in the striatum of mouse brains. Dopaminergic projections in the STR of Ubc9-overexpressing Tg mice (*n* = 7) are significantly protected against MPTP toxicity, compared with WT-MPTP (*n* = 7). ***C***, The number of TH+ neurons in the SNc was significantly higher in Ubc9-Tg mice (*n* = 7) than that in WT siblings (*n* = 7), after chronic MPTP treatment. Dots represent values from individual mouse and horizontal bars represent mean ± SEM. Statistical analysis was applied using two-way ANOVA, Tukey’s test; **p < 0.01, ***p < 0.001, ****p < 0.0001; size bars (500 μm for STR and 100 μm for SNc).

**Figure 5.**
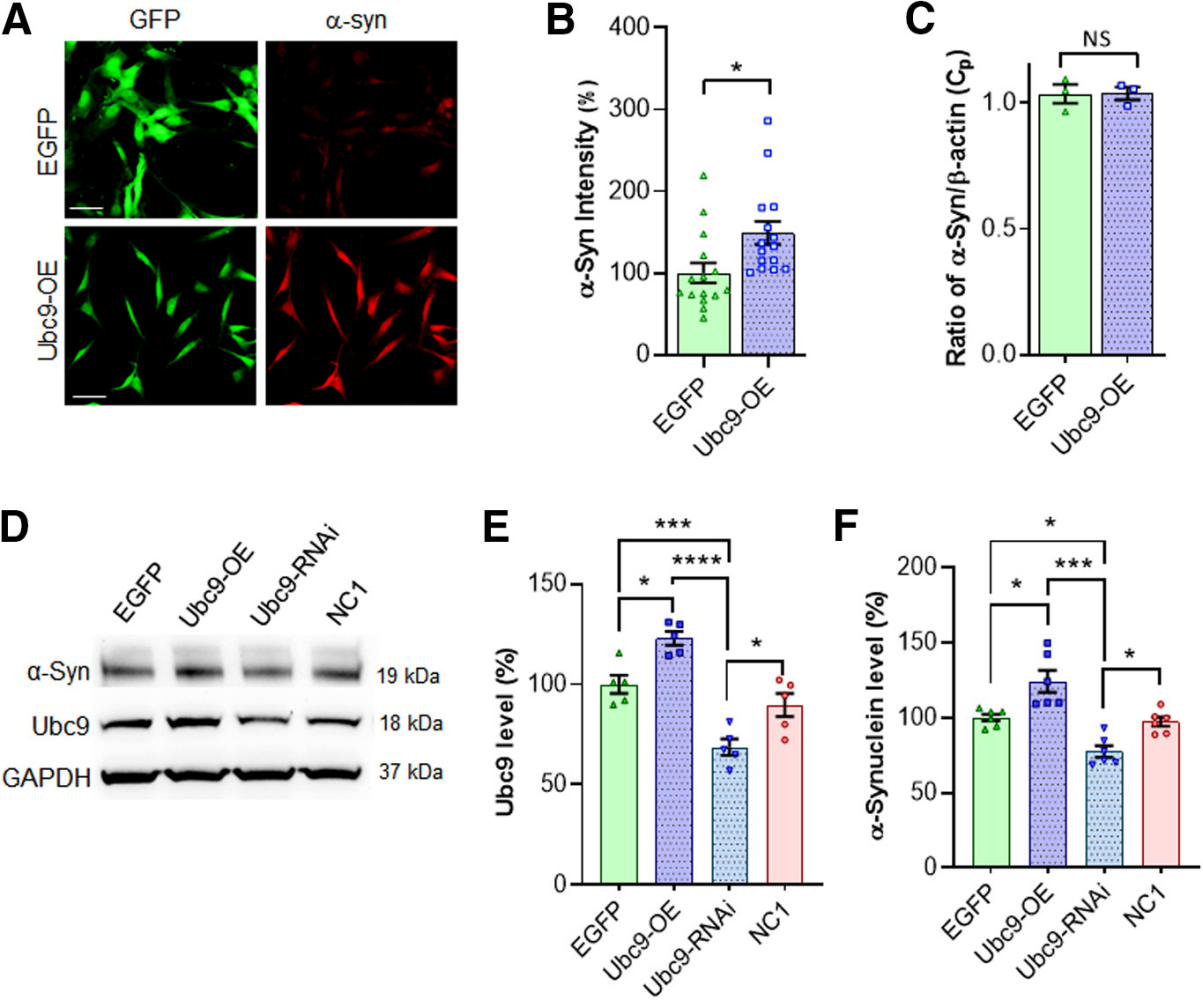
Ubc9 overexpression increases the endogenous level of α-syn protein in dopaminergic N27 cells. ***A***, Immunofluorescent signals for α-syn and EGFP in N27 cell lines stably expressing either Ubc9-EGFP or EGFP, are shown as indicated. Image scale bar: 20 μm. ***B***, In the quantification of fluorescence intensity of endogenous α-syn in immunocytochemistry, higher level of α-syn was detected in Ubc9 cells than in EGFP cells (*n* = 15). Statistical analysis was applied to Student’s unpaired *t* test. ***C***, In the qRT-PCR, there is no difference in mRNA level of α-syn between Ubc9 and EGFP cells (*n* = 3 × 3). ***D***, In WBs, the levels of α-syn and Ubc9 are displayed with the treatment of Ubc9-OE or -RNAi, in comparison to EGFP and NC1 controls. ***E***, Ubc9 expression was upregulated by Ubc9-OE and downregulated by Ubc9-RNAi in the quantification of WBs (*n* = 5 per group). ***F***, In the analysis of 24-h chase assays, the mixed Ubc9-RNAi constructs significantly reduces the level of α-syn, compared with the control (NC1) in WBs, whereas Ubc9-OE increases the level of α-syn, compared with EGFP control (*n* = 6). Bars represent mean ± SEM, and each dot represents the mean of each experiment, which was performed in triplicate. Statistical analysis was applied to one-way ANOVA, Tukey’s *post hoc* test; *p <0.05, ***p <0.001, ****p < 0.0001; ns: not significant.

**Figure 6.**
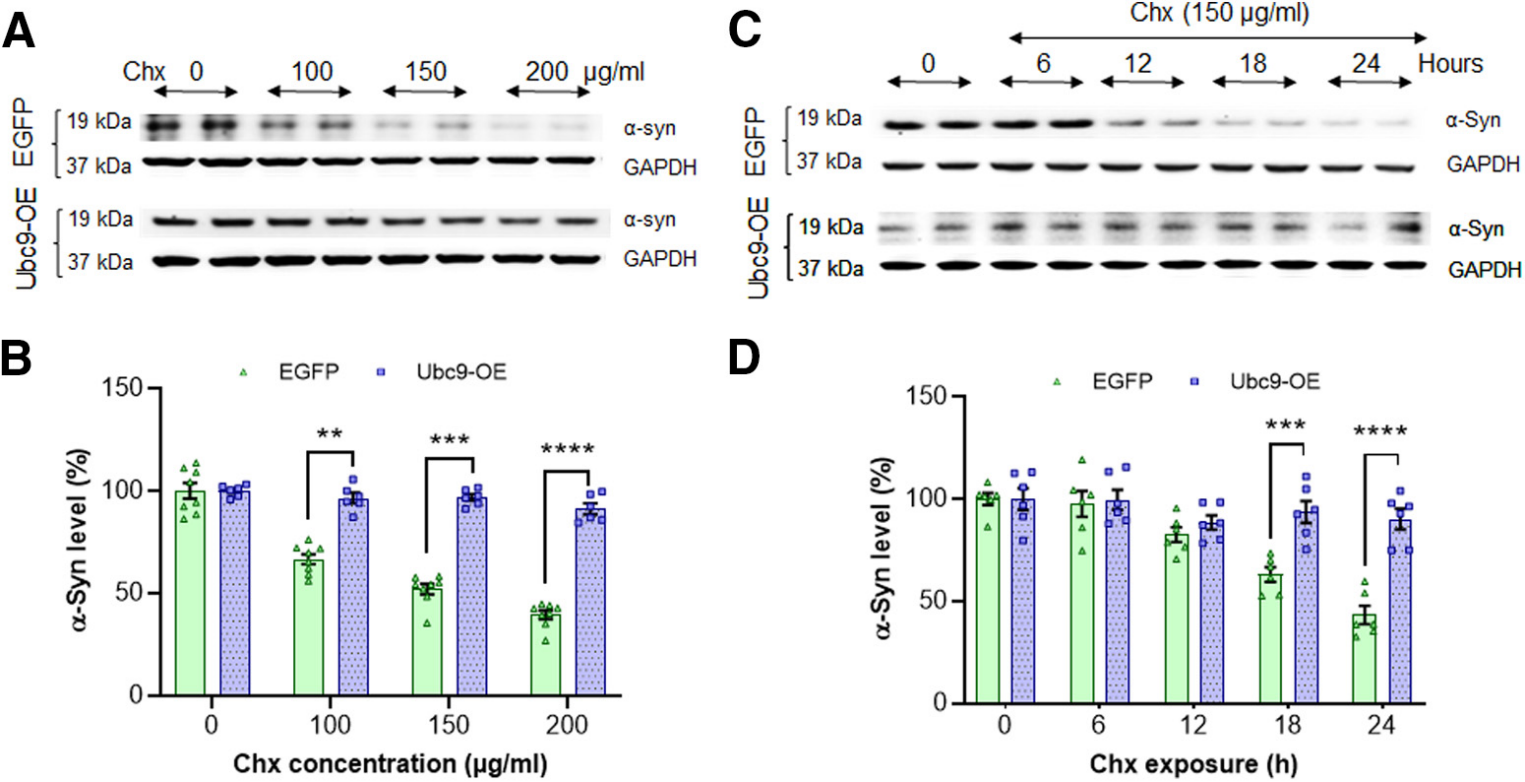
Ubc9 overexpression significantly prevents α-syn degradation, compared with EGFP only in 24-h Chx-treated chase assays. ***A***, In WBs, the degradation of α-syn was delayed by Ubc9 overexpression at different concentrations of Chx for 24 h. ***B***, The protein level of α-syn in N27-EGFP cells declines in the dose-dependent manner of Chx in 24-h chase study, whereas Ubc9-OE prevents the degradation of α-syn. ***C***, In WBs, the degradation of α-syn in EGFP cells was detected in a time-dependent manner at various time exposures of Chx (150 μg/ml). ***D***, In the treatment of 150 μg/ml of Chx, >50% of α-syn was degraded in EGFP cells, while there is no significant decrease in Ubc9-OE cells at different time points up to 24 h. Integrated density of WB images was measured using ImageJ. The ratio of α-syn/GAPDH was presented as mean ± SEM in % of control (*n* = 6–8 each time point). Statistical analysis was applied using two-way ANOVA, Tukey’s *post hoc* test; **p < 0.01, ***p < 0.001, ****p < 0.0001.

**Figure 7.**
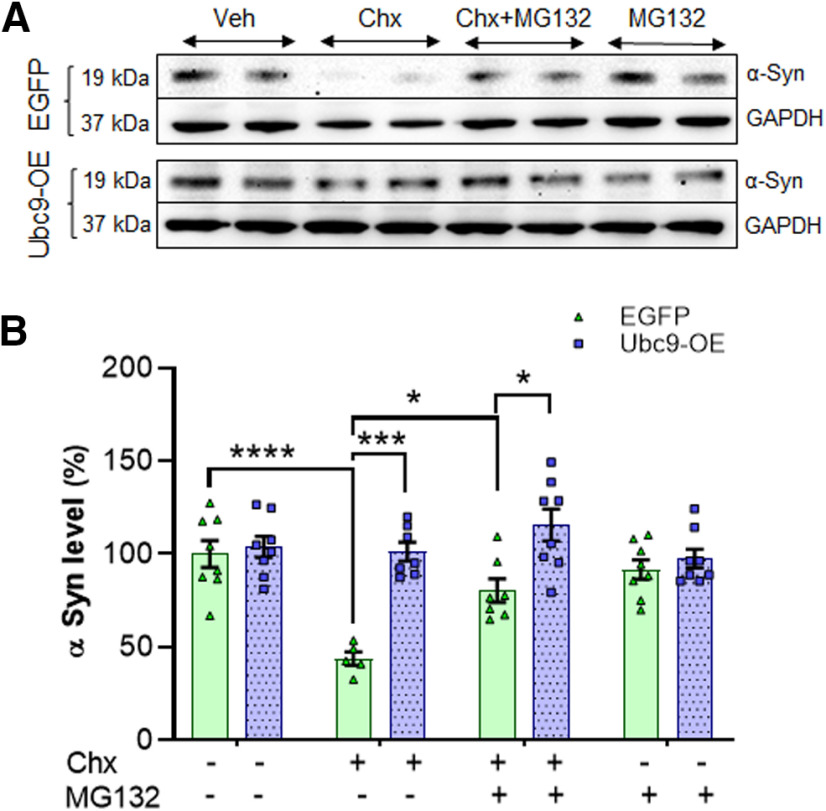
MG132-mediated proteasomal inhibition prevents α-syn degradation in EGFP cells, while it shows no effect in Ubc9 cells in 24-h Chx chase studies. ***A***, In WBs, there is no noticeable effect of MG132 (proteasome inhibitor) in Ubc9 cells, compared with EGFP cells. ***B***, In the analysis of integrated density of WB images, α-syn degradation was significantly interrupted by Ubc9 overexpression compared with EGFP cells. The ratio of α-syn/GAPDH was displayed as mean ± SEM in % of control (*n* = 6–8 each). Two-way ANOVA was applied to assess the interaction between Ubc9 and MG132 for statistical analysis; *p < 0.05, ***p < 0.001, ****p < 0.0001.

**Figure 8.**
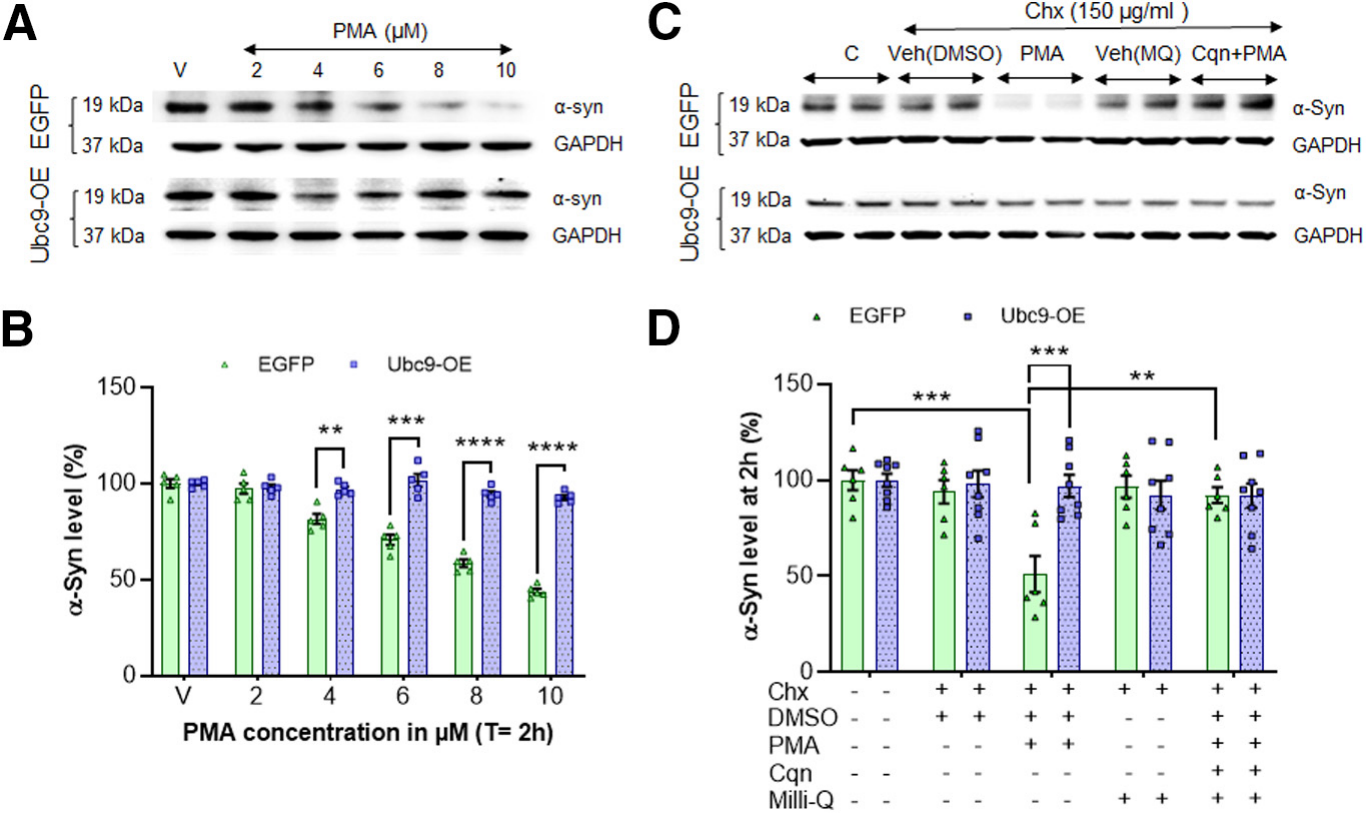
Ubc9 overexpression significantly prevents the PMA-induced lysosomal degradation of α-syn in 2-h protein degradation analysis. ***A***, In WBs, the level of α-syn in N27-EGFP cells declines in the dosage-dependent manner of PMA treatment in 2-h chase study, while that in Ubc9-OE cells remains the same. ***B***, In the integrated density analysis of WB images, Ubc9-OE significantly prevents PMA-induced degradation of α-syn even at 10 μm PMA (*n* = 5). ***C***, After the optimization of PMA and Chx at various concentrations, 5 μm PMA and 150 μg/ml of Chx were applied to EGFP and Ubc9 cells for WBs. ***D***, In the integrated density analysis of WB images, PMA in EGFP cells substantially induces the degradation of α-syn in 24-h chase study, while Ubc9 overexpression prevents the PMA-induced lysosomal degradation of α-syn. No treatment control (***C***) was considered as 100% in the relative ratio of α-syn/GAPDH and DMSO was vehicle for PMA treatment. The second vehicle for the PMA inhibitor, chloroquine (Cqn) was distilled water (MQ, Milli-Q H_2_O), which was included as well as Cqn+PMA for comparison. The ratio of α-syn/GAPDH was presented in mean ± SEM in % of control (*n* = 5–8 per treatment). Statistical analysis was performed by using two-way ANOVA to assess the interaction between Ubc9 and PMA; **p <0.01, ***p < 0.001, ****p < 0.0001.

**Figure 9.**
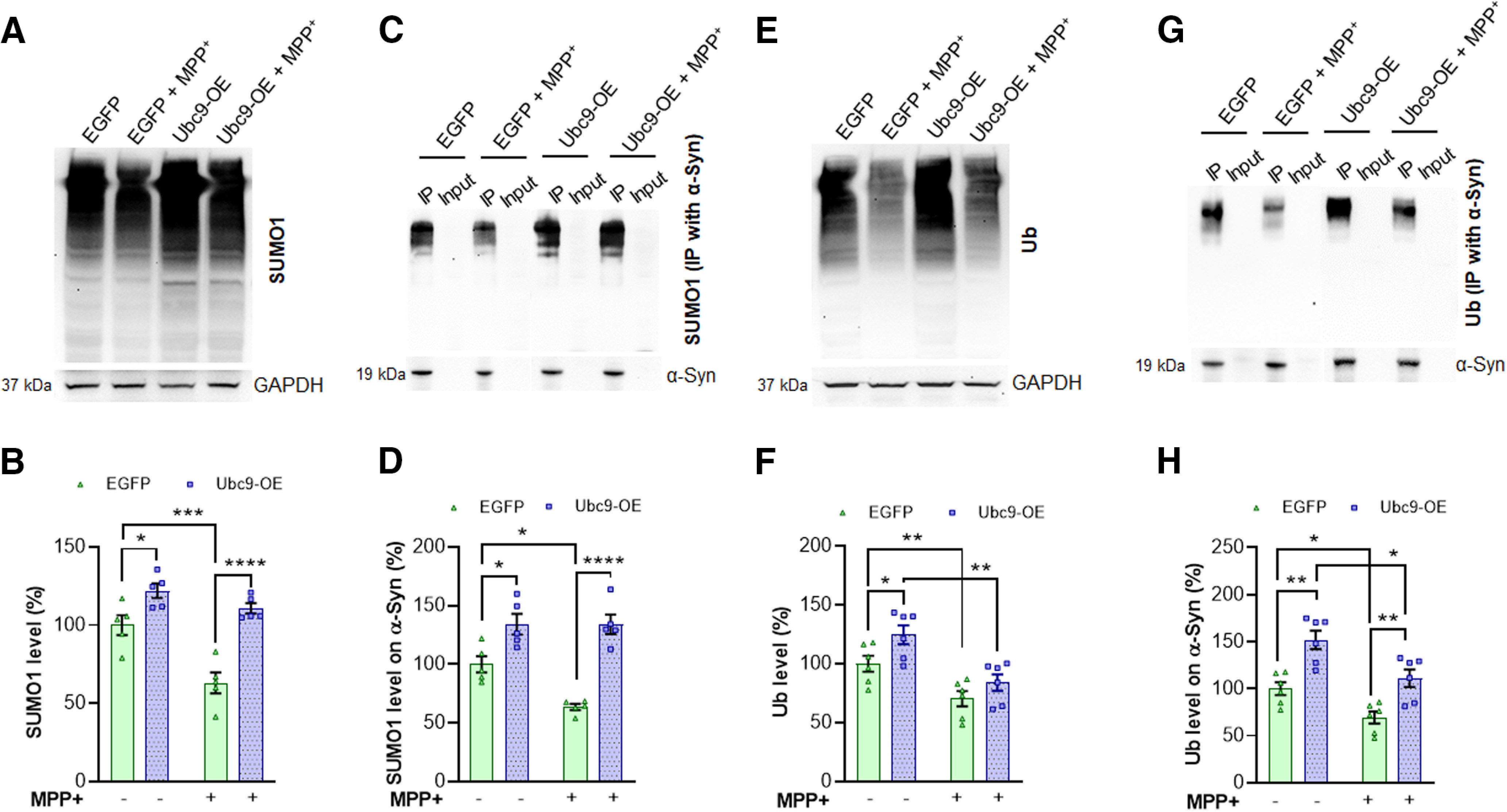
Ubc9 overexpression enhances the levels of SUMO1 and ubiquitin on α-syn, while it prevents MPP+-induced SUMO-1 removal from α-syn. ***A***, WB images show that the level of SUMO1 was increased with Ubc9 overexpression from total cell lysates. GAPDH was used as a loading control for total cell lysates. ***B***, MPP+ exposure significantly reduces the total amount of SUMO1 in N27-EGFP cells, while Ubc9-OE prevents MPP+-induced SUMO1 down-regulation in total cell lysates. ***C***, In WB using IP’ed α-syn, MPP+ reduces the level of SUMO1 bound to α-syn in EGFP cells. α-Syn was used as a loading control after IPs. ***D***, Ubc9 overexpression prevents MPP+-induced SUMO1 removal from α-syn in quantitative WB analysis. ***E***, Examples of WB show that the total amount of ubiquitin (Ub) was reduced by MPP+ treatment. GAPDH was used as a loading control for total cell lysates. ***F***, The levels of ubiquitin from total cell lysates were decreased by MPP+ exposure both in EGFP and Ubc9 cells. ***G***, In WB, the levels of ubiquitin bound to α-syn were substantially reduced by MPP+ treatment. α-Syn was used as a loading control after IPs. ***H***, MPP+ exposure results in a significant reduction of ubiquitin bound to α-syn in both EGFP and Ubc9 cells. MPP+ (640 μm) treatment for 24 h was applied to all experiments. Integrated density of WB images was measured using ImageJ. The ratios of SUMO1 or ubiquitin over respected loading control were presented as mean ± SEM in % of control. Statistical analysis was applied using two-way ANOVA, Tukey’s multiple comparison *post hoc* test; *p < 0.05, **p < 0.01, ***p < 0.001, ****p < 0.0001 (*n* = 5–8 each group).

In most statistical analyses, two-way ANOVA was applied to assess the potential interaction between Ubc9-OE (or Ubc9-RNAi) and toxic treatment (MPP+ or PFF) in Figures 1-4, 9. In Figures 6-8, two-way ANOVA, Tukey’s multiple comparison was also applied to analyze the interaction between Ubc9 and MG132 (or PMA), related to protein degradation. Student’s unpaired *t* test was applied to assess the Ubc9 effects, compared with EGFP only treatment (Fig. 5*B*,*C*). One-way ANOVA, Tukey’s *post hoc* test was applied to Figure 5*E*, *F*. In Extended Data Figure 1-1, one-way ANOVA, Dunnett’s test was adopted to compare different concentrations of treatment with vehicle control. For all studies, *p* < 0.05 was considered statistically significant (*); GraphPad Prism 8.03 software was used for all data analyses and display. Values are presented as mean ± SEM.

10.1523/ENEURO.0134-20.2020.f1-1 Extended Data Figure 1-1 The optimal toxic dose of α-syn PFF in N27 parental cells was assessed in the range of 1–5 μg/ml for cell viability (MTT; ***A***) and cytotoxicity (LDH; ***B***) assays. Our tests indicate that 1 μg/ml of PFF was consistently toxic to N27 cells, compared to vehicle-treated control (green bar) in both MTT and LDH assays. Scattered dot plots represent mean ± SEM (*n* = 8–16). One-way ANOVA, Dunnett’s test was applied for significance and vehicle was depicted for comparison; ****p < 0.0001. Download Figure 1-1, TIF file.

## Results

### Cell viability and cytotoxicity

MPP+ treatment to both EGFP and Ubc9-EGFP cells decreased mitochondrial dehydrogenase activity with a gradient. After 24 h of MPP+ treatment at various concentrations (up to 1280 μm), cell viability in EGFP cells declined sharply in a dose-dependent manner, while that in Ubc9 cells decreased gradually compared with their vehicle groups. Ubc9 overexpression significantly ameliorated the toxic effects at all concentrations of MPP+ compared with EGFP cells (Fig. 1*A*). Ubc9 cells showed significantly higher cell viability in terms of mean differences than EGFP cells at all the concentrations of MPP+ (160–1280 μm; Fig. 1*A*; Table 1). In LDH assays, MPP+ treatment also increased cytotoxicity in both EGFP and Ubc9 cells as estimated by the release of cytosolic enzyme LDH in culture medium. The LDH activity in the vehicle group of EGFP and Ubc9 cells was calculated as 100% of control. After 24 h of MPP+ treatment at various concentrations, cytotoxicity in EGFP cells was gradually elevated in a dose-dependent manner. In comparison with EGFP cells, Ubc9 cells showed 50.91% (*p* < 0.0001) and 41.09% (*p* < 0.0001) less cytotoxicity in mean differences at the concentrations of 320 and 640 μm MPP+, respectively. However, there were no significant differences at 160 and 1280 μm MPP+, probably because of too little or too much LDH release by MPP+ at the concentration, respectively (Fig. 1*B*; Table 1).

**Table 1:** Statistical analyses in **Figures 1–4**

Location	Data structure	Type of test	CI/power	*P* value	Comparison
1*A*	Normally distributed	Two-way ANOVA	–21.49 to –13.89	<0.0001	Cell viability after MPP+ treatment
	Normally distributed	Tukey's *post hoc*	–38.98 to –11.11	<0.0001	Ubc9-OE 160 vs EGFP 160
	Normally distributed	Tukey's *post hoc*	–43.29 to –15.41	<0.0001	Ubc9-OE 320 vs EGFP 320
	Normally distributed	Tukey's *post hoc*	–33.00 to –5.130	0.0012	Ubc9-OE 640 vs EGFP 640
	Normally distributed	Tukey's *post hoc*	–28.93 to –1.055	0.0251	Ubc9-OE 1280 vs EGFP 1280
1*B*	Normally distributed	Two-way ANOVA	10.85 to 24.22	<0.0001	Cytotoxicity after MPP+ treatment
	Normally distributed	Tukey's *post hoc*	–21.26 to 27.73	>0.9999	Ubc9-OE 160 vs EGFP 160
	Normally distributed	Tukey's *post hoc*	26.41 to 75.40	<0.0001	Ubc9-OE 320 vs EGFP 320
	Normally distributed	Tukey's *post hoc*	16.59 to 65.58	<0.0001	Ubc9-OE 640 vs EGFP 640
	Normally distributed	Tukey's *post hoc*	–32.05 to 16.94	0.9909	Ubc9-OE 1280 vs EGFP 1280
1*C*	Normally distributed	Two-way ANOVA	13.94 to 24.49	0.1975	Cell viability after RNAi and MPP+ treatment
	Normally distributed	Tukey's *post hoc*	4.795 to 43.66	0.005	Ubc9-OE 320 vs EGFP 320
	Normally distributed	Tukey's *post hoc*	10.08 to 48.95	0.0003	Ubc9-OE 640 vs EGFP 640
	Normally distributed	Tukey's *post hoc*	4.632 to 43.50	0.0054	Ubc9-OE 1280 vs EGFP 1280
1*D*	Normally distributed	Two-way ANOVA	–232.5 to –145.8	<0.0001	Cytotoxicity after RNAi and MPP+ treatment
	Normally distributed	Tukey's *post hoc*	–345.3 to –25.87	0.0115	Ubc9-OE 320 vs EGFP 320
	Normally distributed	Tukey's *post hoc*	–391.8 to –72.35	0.0006	Ubc9-OE 640 vs EGFP 640
	Normally distributed	Tukey's *post hoc*	–634.4 to –314.9	<0.0001	Ubc9-OE 1280 vs EGFP 1280
1*E*	Normally distributed	Two-way ANOVA	–12.63 to –4.563	<0.0001	Cell viability after PFF treatment
	Normally distributed	Tukey's *post hoc*	–23.71 to –2.991	0.0041	Ubc9-OE WGA/Glu vs EGFP WGA/Glu
	Normally distributed	Tukey's *post hoc*	–22.63 to –2.255	0.0077	Ubc9-OE PFF vs EGFP PFF
1*F*	Normally distributed	Two-way ANOVA	28.05 to 35.19	<0.0001	Cytotoxicity after PFF treatment
	Normally distributed	Tukey's *post hoc*	23.77 to 41.89	0.0001	Ubc9-OE WGA/Glu vs EGFP WGA/Glu
	Normally distributed	Tukey's *post hoc*	52.98 to 71.09	<0.0001	Ubc9-OE PFF vs EGFP PFF
1*G*	Normally distributed	Two-way ANOVA	4.118 to 18.39	<0.0001	Cell viability after Ubc9-RNAi knockdown
	Normally distributed	Tukey's *post hoc*	–20.35 to 16.00	0.9993	Ubc9-RNAi WGA/Glu vs NC1 WGA/Glu
	Normally distributed	Tukey's *post hoc*	16.56 to 52.91	<0.0001	Ubc9-RNAi PFF vs NC1 PFF
1*H*	Normally distributed	Two-way ANOVA	–55.73 to –27.40	0.0005	Cytotoxicity after Ubc9-RNAi knockdown
	Normally distributed	Tukey's *post hoc*	–69.11 to 3.038	0.0914	Ubc9-RNAi WGA/Glu vs NC1 WGA/Glu
	Normally distributed	Tukey's *post hoc*	–117.0 to –44.85	<0.0001	Ubc9-RNAi PFF vs NC1 PFF
2*B*	Normally distributed	Two-way ANOVA	312.0 to 507.6	<0.0001	ROS level after MPP+ treatment
	Normally distributed	Tukey's *post hoc*	–1460 to –1091	<0.0001	EGFP V vs EGFP MPP+
	Normally distributed	Tukey's *post hoc*	–592.4 to –222.7	0.0025	Ubc9-OE V vs Ubc9-OE MPP+
	Normally distributed	Tukey's *post hoc*	659.0 to 1029	<0.0001	EGFP MPP+ vs Ubc9-OE MPP+
2*D*	Normally distributed	Two-way ANOVA	–434.9 to –212.1	0.0017	ROS levels after RNAi and MPP+ treatment
	Normally distributed	Tukey's *post hoc*	–414.7 to –124.7	0.001	NC1 V vs NC1 MPP+
	Normally distributed	Tukey's *post hoc*	–827.0 to –417.7	<0.0001	Ubc9-RNAi V vs Ubc9-RNAi MPP+
	Normally distributed	Tukey's *post hoc*	–708.2 to –304.4	0.0001	NC1 MPP+ vs Ubc9-RNAi MPP+
2*F*	Normally distributed	Two-way ANOVA	–465.1 to –244.0	<0.0001	ROS level after MPP+ treatment
	Normally distributed	Tukey's *post hoc*	–803.4 to –479.8	<0.0001	EGFP WGA/Glu vs EGFP PFF
	Normally distributed	Tukey's *post hoc*	238.0 to 654.2	<0.0001	EGFP PFF vs Ubc9-OE PFF
2*H*	Normally distributed	Two-way ANOVA	–789.3 to –556.9	<0.0001	ROS levels after RNAi and PFF treatment
	Normally distributed	Tukey's *post hoc*	–510.4 to –79.65	0.0042	NC1 WGA/Glu vs NC1 PFF
	Normally distributed	Tukey's *post hoc*	–1273 to –829.1	<0.0001	NC1 PFF vs Ubc9-RNAi PFF
	Normally distributed	Tukey's *post hoc*	–1062 to –631.3	<0.0001	Ubc9-RNAi WGA/Glu vs Ubc9-RNAi PFF
3*B*	Normally distributed	Two-way ANOVA	–151.0 to –87.73	0.0039	ThioflavinT staining after PFF treatment
	Normally distributed	Tukey's *post hoc*	–203.5 to –53.90	<0.0001	NC1 WGA/Glu vs NC1 PFF
	Normally distributed	Tukey's *post hoc*	–219.6 to –67.66	<0.0001	NC1 PFF vs Ubc9-RNAi PFF
	Normally distributed	Tukey's *post hoc*	–231.4 to –100.4	<0.0001	Ubc9-RNAi WGA/Glu vs Ubc9-RNAi PFF
3*C*	Normally distributed	Two-way ANOVA	–34.85 to –5.685	0.0011	α-Syn staining after PFF treatment
	Normally distributed	Tukey's *post hoc*	–45.05 to 36.34	0.9996	NC1 WGA/Glu vs NC1 PFF
	Normally distributed	Tukey's *post hoc*	–92.75 to –21.75	0.0001	NC1 PFF vs Ubc9-RNAi PFF
	Normally distributed	Tukey's *post hoc*	–94.52 to –22.60	0.0001	Ubc9-RNAi WGA/Glu vs Ubc9-RNAi PFF
4*B*	Normally distributed	Two-way ANOVA	–25.95 to –8.554	<0.0001	TH staining in striatum
	Normally distributed	Tukey's *post hoc*	43.82 to 76.84	<0.0001	Saline WT vs MPTP WT
	Normally distributed	Tukey's *post hoc*	37.62 to 70.64	<0.0001	Saline Ubc9-OE vs MPTP WT
	Normally distributed	Tukey's *post hoc*	–55.78 to –25.64	0.0001	MPTP WT vs MPTP Ubc9-OE
4*C*	Normally distributed	Two-way ANOVA	–1522 to –383.4	0.0082	TH positive cells in substantia nigra
	Normally distributed	Tukey's *post hoc*	1002 to 3163	0.0021	Saline WT vs MPTP WT
	Normally distributed	Tukey's *post hoc*	1154 to 3315	0.0002	Saline Ubc9-OE vs MPTP WT
	Normally distributed	Tukey's *post hoc*	–2741 to –767.7	0.0040	MPTP WT vs MPTP Ubc9-OE

Next, we applied various concentrations of MPP+ as above (Fig. 1*A*,*B*) to Ubc9-RNAi-treated N27 cells for measuring cell viability and cytotoxicity. In MTT assays (Fig. 1*C*), we found that RNAi-mediated Ubc9 knock-down significantly exacerbated the MPP+-induced toxicity in N27 cells at 320, 640, and 1280 μm MPP+ (Table 1), compared with the random RNAi constructs control (NC1 from IDT). In LDH assays (Fig. 1*D*), we found the same patterns as cell viability assays that Ubc9-RNAi significantly enhanced the MPP+-mediated toxicity at 320, 640, and 1280 μm MPP+ (Table 1), compared with the NC1 control. Based on Figure 1*A–D*, 640 μm MPP+ was applied to additional experiments.

We also exposed N27 cells to the PFFs of α-syn to assess the protective effect of Ubc9-mediated SUMOylation in MTT and LDH assays. First, we tested the PFF exposure to N27 cells in the range of 1–5 μg/ml to determine the optimal dose and demonstrate in the Extended Data Figure 1-1 that 1 μg/ml of PFF treatment was sufficient to induce significant toxicity *in vitro* as reported (Volpicelli-Daley et al., 2011; Polinski et al., 2018). Next, using 1 μg/ml of PFF treatment, we tested the effects of Ubc9 overexpression or knock-down against PFF toxicity. Our results indicate that the overexpression of Ubc9 mitigates the PFF-induced toxicity compared with EGFP control cells. In Figure 1*E*, some level of cell damage was observed from WGA/GluNAc only (PFF penetration mediator/blocker), but Ubc9 overexpression significantly protected N27 cells from PFF-induced toxicity in both cell viability (*p* = 0.0077; Fig. 1*E*) and cytotoxicity assays (*p* < 0.0001; Fig. 1*F*). In the following experiments, we applied PFF exposure to Ubc9-RNAi-treated N27 cells and found that RNAi-mediated Ubc9 knock-down significantly enhanced the PFF-induced toxicity in N27 cells in both cell viability (*p* < 0.0001; Fig. 1*G*) and cytotoxicity (*p* < 0.0001; Fig. 1*H*), compared with the random RNAi constructs control (NC1 from IDT).

### ROS levels

MPP+ (640 μm) exposure for 24 h caused a drastic increase in ROS generation in both EGFP and Ubc9-EGFP cells. The base level of ROS increased 13.76-fold (*p* < 0.0001) in EGFP cells, whereas it increased 4.28-fold in Ubc9 cells (*p* = 0.0025) by 640 μm MPP+ (Fig. 2*A*,*B*). Thus, Ubc9 overexpression significantly suppressed MPP+-induced ROS generation when compared with EGFP only (*p *<* *0.0001; Fig. 2*B*). In the following experiments, we exposed Ubc9-RNAi to N27 parental cells overnight before MPP+ treatment and then measured the level of ROS. We also found that Ubc9 knock-down enhanced MPP+-induced ROS generation almost 2.5-fold higher than NC1 control (*p* < 0.0001; Fig. 2*C*,*D*). In addition, we compared the level of ROS between Ubc9-overexpressing cells and EGFP cells after PFF (1 μg/ml) exposure for 24 h (Fig. 2*E*). PFF treatment increased ∼7-fold higher ROS generation than the WGA/GluNAc control in EGFP cells (*p* < 0.0001), while it did not significantly increase ROS level in Ubc9-OE cells, compared with the WGA/GluNAc control. Thus, PFF-induced ROS generation was substantially suppressed by Ubc9 overexpression compared with EGFP controls (*p* < 0.0001; Fig. 2*F*). Furthermore, we measured the level of ROS with Ubc9-RNAi and confirmed that Ubc9 knock-down robustly enhanced PFF-induced ROS generation compared with NC1 (PFF-treated group; *p* < 0.0001; Fig. 2*G*,*H*). Although the PFF penetration accommodating reagent/blocker (WGA/GluNAc) increased ROS level subtly, compared with no treatment control, it was not statistically significant (data not shown). These results strongly support that Ubc9-mediated pan-SUMOylation reduces both MPP+-mediated and PFF-induced ROS production *in vitro,* explaining, at least in part, the protective effects by Ubc9-mediated SUMOylation shown in Figure 1.

### Ubc9 knock-down by RNAi enhances PFF-induced protein aggregation in thioflavin T staining

In order to assess the mechanisms of cellular protection by Ubc9 from PFF-induced toxicity, we also measured the level of protein aggregation, which is proportionally labeled in thioflavin T staining (Fig. 3*A*). As a PD model system, the PFF inoculation (1 μg/ml) triggers thioflavin T-labeled protein aggregation in which α-syn was detected (Fig. 3*A*, bottom row, merged). When N27 parental cells with NC1 were exposed to PFF treatment, a low level of protein aggregation was consistently detected in thioflavin T staining (Fig. 3*A*, second row-third column). However, Ubc9-RNAi treatment aggravates the protein aggregation, which was clearly detected in thioflavin T staining (Fig. 3*A*, second row-sixth column). In quantitative analysis, PFF treatment resulted in protein aggregation in N27 parental cells with NC1, and Ubc9 knock-down by RNAi significantly enhanced the level of protein aggregation detected by thioflavin T stain, compared with the NC1/WGA-GluNAc control treatment (*p* < 0.0001; Fig. 3*B*). In addition, we also found that PFF treatment increased the level of α-syn in protein aggregates with Ubc9-RNAi (*p* = 0.0001; Fig. 3*C*) and the treatment of Ubc9-RNAi increased the vulnerability of PFF-induced α-syn accumulation in protein aggregates (*p* < 0.001; Fig. 3*C*). However, the level of α-syn in thioflavin T-stained protein aggregates in NC1 was not significantly higher with PFF based on our analysis (Fig. 3*C*).

### Pan-Ubc9 overexpression protects dopaminergic neurons in the striatum and SNc from MPTP toxicity

Coronal sections of mouse brain were stained by using anti-TH antibody (1:100) to detect dopaminergic neurons. MPTP exerts deleterious effects on dopaminergic neurons of WT C57Bl/6 mice (Lazzara et al., 2015). Immunohistochemistry results showed a significant (*p* < 0.0001) decrease in TH+ dopaminergic neuronal projections in the striatum region of MPTP-treated WT mice when compared with saline-treated WT or Ubc9 transgenic mice (Fig. 4*A*). After the confirmation of pan-Ubc9 overexpression in brain from Ubc9-Tg mice in Western blottings (WBs; Lee et al., 2011), we found that the hemizygous Ubc9-Tg mice showed significant (*p* = 0.0001) protection in the striatum from MPTP toxicity, compared with a WT sibling group in the measurement of TH+ density (Fig. 4*A*,*B*). Similarly, dopaminergic cell body count in the SNc was significantly reduced in MPTP-treated WT mice, compared with saline treated WT (*p* = 0.0021) or Ubc9 groups (*p* = 0.0002). However, Ubc9-Tg mice showed significant (*p* = 0.004) protection against MPTP-induced deleterious effects compared with MPTP-treated WT mice, in the analysis of two-way ANOVA, Tukey’s test (Fig. 4*A*,*C*).

### Endogenous levels of α-syn

The SUMO conjugase Ubc9 is the main step to transfer SUMO1 and SUMO2/3 to target proteins. Therefore, we tested the effects of Ubc9 overexpression on endogenous levels of α-syn. As shown in Figure 5*A*, Ubc9 overexpression in N27 cells increased the endogenous level of α-syn almost 49% when compared with EGFP cells in immunocytochemistry (*p* = 0.0142; Fig. 5*B*). The endogenous mRNA level of α-syn remained unchanged even with Ubc9 overexpression (Fig. 5*C*). In WB analyses after overnight cultures, we demonstrate that the protein level of Ubc9 was ∼25% upregulated by Ubc9 overexpression compared with EGFP control (*p* = 0.012) and almost 40% downregulated by Ubc9-RNAi compared with random cocktail controls (NC1; *p* = 0.0229; Fig. 5*D*,*E*). In the quantification of α-syn WBs, Ubc9 overexpression increased almost 25% of α-syn protein level compared with EGFP control (*p* = 0.025), while the combined Ubc9 knock-down constructs substantially reduced α-syn protein compared with NC1 control (*p* = 0.029; Fig. 5*F*). This result indicates that the enhanced level of α-syn protein in Ubc9 cells is not derived from the increased mRNA level of α-syn, suggesting that SUMOylated α-syn avoids normal protein degradation as it was demonstrated in the DAT study (Cartier et al., 2019).

### Ubc9 overexpression reduces α-syn degradation rate

In Figure 5*B*, we demonstrate that Ubc9 overexpression enhanced the protein level of α-syn, which was not derived from transcriptional up-regulation (Fig. 5*C*). We then tested to see whether Ubc9-induced SUMOylation prevents endogenous α-syn degradation in 24 h chase analysis. In order to quantify the level of the protein degradation, we performed α-syn WBs using different concentrations of the protein synthesis inhibitor, Chx to compare the remaining amount of α-syn in Ubc9 cells with that in EGFP cells. After blocking new protein synthesis, the remaining amount of α-syn treated with vehicle in EGFP cells is considered 100 ± 12.79% as control, and the amount declines as the concentrations of Chx increase. In contrast, the levels of α-syn in Ubc9 cells were barely affected by the increase of Chx concentration (Fig. 6*A*). The degradation rate of α-syn was substantially reduced in Ubc9 cells compared with EGFP cells, resulting in significantly higher levels of α-syn in Ubc9 cells than in EGFP cells at 100, 150, and 200 μg/ml of Chx (Fig. 6*B*; Table 2).

**Table 2:** Statistical analyses in **Figures 5-9 and Extended Data Figure 1-1**

Location	Data structure	Type of test	CI/power	*P* value	Comparison
5*B*	Normally distributed	Unpaired *t* test	10.63 to 87.29	0.0142	EGFP vs Ubc9-OE
5*E*	Normally distributed	One-way ANOVA	0.8219	<0.0001	Ubc9 expression levels
	Normally distributed	Tukey's *post hoc*	–41.45 to –4.625	0.012	EGFP vs Ubc9-OE
	Normally distributed	Tukey's *post hoc*	12.86 to 49.68	0.0009	EGFP vs Ubc9-RNAi
	Normally distributed	Tukey's *post hoc*	35.89 to 72.71	<0.0001	Ubc9-OE vs Ubc9-RNAi
	Normally distributed	Tukey's *post hoc*	–39.38 to –2.560	0.0229	Ubc9-RNAi vs NC1
5*F*	Normally distributed	One-way ANOVA	0.7241	0.0001	α-Syn expression levels
	Normally distributed	Tukey's *post hoc*	–42.22 to –6.078	0.025	EGFP vs Ubc9-OE
	Normally distributed	Tukey's *post hoc*	4.374 to 40.51	0.0117	EGFP vs Ubc9-RNAi
	Normally distributed	Tukey's *post hoc*	28.52 to 64.66	0.0001	Ubc9-OE vs Ubc9-RNAi
	Normally distributed	Tukey's *post hoc*	–37.82 to –1.682	0.029	Ubc9-RNAi vs NC1
6*B*	Normally distributed	Two-way ANOVA	–35.37 to –27.83	<0.0001	Chx chase study at diff concentration
	Normally distributed	Tukey's *post hoc*	–41.70 to –17.93	0.0135	Ubc9-OE 100 vs EGFP 100
	Normally distributed	Tukey's *post hoc*	–56.70 to –32.92	0.0001	Ubc9-OE 150 vs EGFP 150
	Normally distributed	Tukey's *post hoc*	–63.67 to –39.89	<0.0001	Ubc9-OE 200 vs EGFP 200
6*D*	Normally distributed	Two-way ANOVA	–22.99 to –11.24	<0.0001	Chx chase study at diff time point
	Normally distributed	Tukey's *post hoc*	–52.30 to –8.994	0.0008	Ubc9-OE 18h vs EGFP 18h
	Normally distributed	Tukey's *post hoc*	–68.64 to –25.34	<0.0001	Ubc9-OE 24h vs EGFP 24h
7*B*	Normally distributed	Two-way ANOVA	–34.53 to –16.53	0.0002	MG132-mediated proteasomal inhibition
	Normally distributed	Tukey's *post hoc*	25.60 to 86.88	<0.0001	EGFP Veh vs EGFP Chx
	Normally distributed	Tukey's *post hoc*	–88.91 to –25.97	0.0001	EGFP Chx vs Ubc9-OE Chx
	Normally distributed	Tukey's *post hoc*	–68.03 to –5.097	0.0343	EGFP Chx vs EGFP Chx+MG132
	Normally distributed	Tukey's *post hoc*	–62.93 to –7.299	0.0111	EGFP Chx+MG132 vs Ubc9 Chx+MG132
8*B*	Normally distributed	Two-way ANOVA	–24.40 to –19.50	<0.0001	PMA chase study
	Normally distributed	Tukey's *post hoc*	–25.64 to –5.157	0.0012	EGFP 4μM vs Ubc9-OE 4μM
	Normally distributed	Tukey's *post hoc*	–41.26 to –20.77	0.0001	EGFP 6μM vs Ubc9-OE 6μM
	Normally distributed	Tukey's *post hoc*	–46.18 to –25.69	<0.0001	EGFP 8μM vs Ubc9-OE 8μM
	Normally distributed	Tukey's *post hoc*	–59.03 to –38.55	<0.0001	EGFP 10μM vs Ubc9-OE μM
8*D*	Normally distributed	Two-way ANOVA	–17.12 to –1.028	0.0009	Effect of PMA on protein degradation
	Normally distributed	Tukey's *post hoc*	16.06 to 81.81	0.0002	EGFP C vs EGFP PMA
	Normally distributed	Tukey's *post hoc*	–76.68 to –15.19	0.0002	EGFP PMA vs Ubc9-OE PMA
	Normally distributed	Tukey's *post hoc*	–73.98 to –8.239	0.0031	EGFP PMA vs EGFP Cqn+PMA
9*B*	Normally distributed	Two-way ANOVA	0.81	<0.0001	SUMO1 level in total lysate
	Normally distributed	Tukey's *post hoc*	15.06 to 58.91	0.001	EGFP vs EGFP MPP+
	Normally distributed	Tukey's *post hoc*	–43.90 to –0.05988	0.0493	EGFP vs Ubc9
	Normally distributed	Tukey's *post hoc*	–69.67 to –25.83	<0.0001	EGFP MPP+ vs Ubc9 MPP+
9*D*	Normally distributed	Two-way ANOVA	0.81	<0.0001	SUMO1 level in α-syn IP'ed samples
	Normally distributed	Tukey's *post hoc*	7.692 to 65.01	0.0011	EGFP vs EGFP MPP+
	Normally distributed	Tukey's *post hoc*	–62.93 to –5.610	0.0166	EGFP vs Ubc9
	Normally distributed	Tukey's *post hoc*	–99.03 to –41.72	<0.0001	EGFP MPP+ vs Ubc9 MPP+
9*F*	Normally distributed	Two-way ANOVA	0.62	0.0002	Ubiquitin level in total lysate
	Normally distributed	Tukey's *post hoc*	1.485 to 57.87	0.0034	EGFP vs EGFP MPP+
	Normally distributed	Tukey's *post hoc*	–52.79 to 3.595	0.0369	EGFP vs Ubc9
	Normally distributed	Tukey's *post hoc*	12.41 to 68.79	0.0036	Ubc9 vs Ubc9 MPP+
9*H*	Normally distributed	Two-way ANOVA	0.72	<0.0001	Ubiquitin level in α-syn IP'ed samples
	Normally distributed	Tukey's *post hoc*	19.70 to 63.21	0.0081	EGFP vs EGFP MPP+
	Normally distributed	Tukey's *post hoc*	–84.50 to –19.32	0.0013	EGFP vs Ubc9
	Normally distributed	Tukey's *post hoc*	–74.35 to –9.169	0.0092	EGFP MPP+ vs Ubc9 MPP+
	Normally distributed	Tukey's *post hoc*	8.176 to 73.36	0.0111	Ubc9 vs Ubc9 MPP+
1-1*A*	Normally distributed	One-way ANOVA	0.81	<0.0001	Effect of PFF on cell viability
	Normally distributed	Dunnett's *post hoc*	25.90 to 47.02	<0.0001	Vehicle vs PFF 1μg
	Normally distributed	Dunnett's *post hoc*	29.84 to 50.96	<0.0001	Vehicle vs PFF 2μg
	Normally distributed	Dunnett's *post hoc*	37.21 to 55.50	<0.0001	Vehicle vs PFF 3μg
	Normally distributed	Dunnett's *post hoc*	44.66 to 65.78	<0.0001	Vehicle vs PFF 4μg
	Normally distributed	Dunnett's *post hoc*	41.98 to 60.27	<0.0001	Vehicle vs PFF 5μg
1-1*B*	Normally distributed	One-way ANOVA	0.78	<0.0001	Effect of PFF on cytotoxicity
	Normally distributed	Dunnett's *post hoc*	–35.37 to –11.32	<0.0001	Vehicle vs PFF 1μg
	Normally distributed	Dunnett's *post hoc*	–37.83 to –13.78	<0.0001	Vehicle vs PFF 2μg
	Normally distributed	Dunnett's *post hoc*	–50.65 to –29.82	<0.0001	Vehicle vs PFF 3μg
	Normally distributed	Dunnett's *post hoc*	–63.02 to –38.97	<0.0001	Vehicle vs PFF 4μg
	Normally distributed	Dunnett's *post hoc*	–61.21 to –40.38	<0.0001	Vehicle vs PFF 5μg

Based on Figure 6*B*, the 150 μg/ml of Chx was applied to determine the optimal time point from total cell lysates (0–24 h; Fig. 6*C*). In WB analyses (duplicate, *n* = 3 independently), α-syn levels in EGFP cells declined gradually from T = 0 (100 ± 12.99%) to T = 24 h (41.58 ± 2.62%). A significant degradation of α-syn was observed from EGFP cells at T = 18 h (*p* = 0.0033) and T = 24 h (*p* = 0.0007) compared with the level at T = 0 (Fig. 6*D*). There was no significant difference observed in Ubc9 cells after Chx treatment over 24 h. When compared with EGFP cells, the mean difference of the remaining α-syn level in Ubc9 was 36.74% higher (*p* = 0.0008) at T = 18 h and 52.01% higher (*p* < 0.0001) at T = 24 h (Fig. 6*D*). Altogether our results indicate that Ubc9 overexpression reduces the degradation rate of α-syn.

### Testing the mechanism of delayed α-syn degradation by SUMOylation, through proteasomal pathway

In the following experiment, we assessed the mechanisms of delayed α-syn degradation, affected by Ubc9-mediated SUMOylation. We tested to see whether the interruption of endogenous α-syn degradation in Ubc9-overexpressing cells results from the inhibition of the ubiquitin-proteasome system (UPS; Yan et al., 2005). After 24-h exposure of the proteasome inhibitor MG132 (10 μm) in Chx (150 μg/ml)-treated chase studies, the endogenous level of α-syn was measured in both Ubc9 and EGFP cell lines using WBs (Fig. 7*A*). Massive degradation of α-syn was detected after Chx treatment (*p* < 0.0001); this was substantially reversed by MG132 co-treatment (*p* = 0.0343) in EGFP cells (the level of α-syn at 24 h: vehicle = 100 ± 7.25%; Chx = 46.07 ± 10.32%; Chx+MG132 = 75.41 ± 10.55%; MG132 = 91.63 ± 5.20%; Fig. 7*B*). However, in Ubc9-OE cells, there was no significant change in the level of α-syn after Chx and/or MG132 treatment (Fig. 7*B*, *n* = 4 per treatment with duplicates). In our analysis, Ubc9 overexpression alone prevented α-syn degradation by the same level as MG132 proteasomal inhibitor in EGFP control cells. Hence, there was no room to show any additive effect by MG132 or to detect the interaction between Ubc9 and MG132 in two-way ANOVA analysis. Therefore, we decided to test the effects of Ubc9 on α-syn degradation via the lysosomal pathway.

### Ubc9 overexpression blocks the lysosomal degradation of α-syn induced by PMA treatment

α-Syn is known to be degraded by UPS and the autophagy-lysosome pathway (ALP); however, it is more likely degraded inside lysosomes through chaperone-mediated autophagy (CMA) or endocytosis (Webb et al., 2003; Cuervo et al., 2004; Martinez-Vicente and Vila, 2013). Therefore, we assessed the capacity of Ubc9 overexpression to impair PMA-induced α-syn degradation through lysosomal degradation (Cartier et al., 2019). We performed Chx chase analyses in the presence of PMA (PKC activator for inducing lysosomal degradation) with or without chloroquine, a known lysosomotropic inhibitor that reduces lysosomal protease activities (Lu et al., 2017). In quantitative WB analysis, we measured how much Ubc9 overexpression prevents the PKC-mediated lysosomal degradation of α-syn. In Figure 8*A*,*B*, we assessed the optimal concentration of PMA in the range of 0–10 μm and confirmed that PMA induced lysosomal degradation of α-syn in EGFP cells in a dose-dependent manner. However, there was no significant effect of PMA in Ubc9 cells (Fig. 8*B*). Based on the results in Figure 8*B*, we found that 5 μm PMA for 2 h was sufficient to trigger lysosomal degradation of α-syn in EGFP cells. The PKC activation by PMA (5 μm) for 2 h induced a significant α-syn degradation in EGFP cells (*p *=* *0.0002), compared with vehicle only (α-syn level: vehicle = 93.92 ± 6.19%; PMA= 51.06 ± 9.43%; chloroquine+PMA= 92.17 ± 4.15%; Fig. 8*D*). The lysosomal degradation of α-syn induced by PMA was blocked by co-incubation with the lysosomal protease inhibitor, chloroquine (*p* = 0.0031). The residual level of α-syn in Ubc9 cells was not significantly affected by PMA treatment because of the robust prevention of α-syn degradation by Ubc9 overexpression (Fig. 8*C*,*D*). Therefore, the interaction between Ubc9 and PMA or the additive effect by PMA was not detected in two-way ANOVA, suggesting that Ubc9-mediated SUMOylated α-syn almost completely avoids PMA-induced lysosomal degradation.

### Ubc9 overexpression prevents the MPP+-induced SUMO1 removal from α-syn

In the following experiments, we assessed the regulatory mechanisms of α-syn by SUMO1 or ubiquitin. In Figure 9*A*, we detected robust expressions of SUMO1 from total cell lysates and found that Ubc9 overexpression increased the total amount of SUMO1 expression compared with EGFP cells (*p* = 0.0493), as expected. When N27 cells were exposed to MPP+, the level of SUMO1 was significantly reduced in EGFP cells (*p* = 0.001), but not decreased in Ubc9-overexpressing cells (ns; Fig. 9*B*). Thus, the level of SUMO1 was significantly higher in Ubc9 cells than that in EGFP cells (*p* < 0.0001; Fig. 9*B*). As expected, we found streaked band patterns even with IP’ed α-syn, probably because of various forms of SUMO1 binding to α-syn (Fig. 9*C*). After α-syn was IP’ed, we found the same pattern that SUMO1 was removed from α-syn by MPP+ in EGFP cells (*p* = 0.0011), in contrast, the level of SUMO1 on α-syn in Ubc9-OE cells was not affected by MPP+ (Fig. 9*D*). Similar to Figure 9*B*, the level of SUMO1 on α-syn in Ubc9 cells was significantly higher than that in EGFP cells (*p* < 0.0001; Fig. 9*D*).

According to the mass spectrometry-based relative quantification of protein interaction with IP’ed α-syn, the level of ubiquitin on α-syn was 2.2-fold higher in Ubc9 cells than in EGFP cells (Extended Data Figure 9-1). Thereafter, we measured the levels of ubiquitin in total cell lysates as well as α-syn IP’ed samples using WBs. Although the level of ubiquitin was higher from Ubc9 cell lysates than that from EGFP cell lysates without MPP+ exposure, MPP+ significantly reduced the total level of ubiquitin in EGFP cells (*p* = 0.0034) as well as in Ubc9 cells (Fig. 9*E*,*F*; Table 2). Similarly, the level of ubiquitin in α-syn IP’ed samples was noticeably reduced by MPP+ exposure in both EGFP and Ubc9 cells. However, the base level of ubiquitin-bound α-syn was substantially higher in Ubc9 cells than in EGFP cells, and the pattern remained the same with MPP+ treatment (Fig. 9*G*,*H*; Table 2). Taken together, these results suggest that Ubc9 overexpression enhances not only the level of SUMO1 on α-syn, but it also increases the level of ubiquitin on α-syn. However, Ubc9 overexpression prevents the MPP+-induced SUMO1 removal from α-syn, while it does not affect the ubiquitin removal from α-syn.

10.1523/ENEURO.0134-20.2020.f9-1 Extended Data Figure 9-1 Ubc9 overexpression increases several proteins’ interactions with α-syn in relative protein level analysis using mass spectrometry. ***A***, The Venn diagram in the analysis of IPe’d α-syn from N27-EGFP and Ubc9-OE cell lysates shows that Ubc9-OE increases protein interaction with α-syn from 208 proteins, while Ubc9-OE decreases 102 proteins’ interactions with α-syn compared with EGFP cells. ***B***, Several proteins in the list including ubiquitin were identified to interact with α-syn >2-fold higher in Ubc9 cells than in EGFP cells. ***C***, A potential protein interaction schematic suggests that α-syn interacts with ubiquitin (Ubb and Uba52) 2.2-fold higher in Ubc9-OE than in EGFP only. ***D***, Some protein interactions are considered as “known” and others are “predicted,” based on potential protein-protein interaction database (UniProt). Download Figure 9-1, TIF file.

## Discussion

In this study, we demonstrate that Ubc9 overexpression protected dopaminergic cells from MPP+- or PFF-induced toxicity *in vitro* and further, pan-Ubc9 overexpression prevented dopaminergic neuronal loss in the striatum and SNc from MPTP-induced toxicities. The mechanism of SUMO-mediated neuroprotection may be, at least in part, derived from the prevention of ROS generation induced by MPP+ or PFF (Fig. 2). In addition, PFF treatment enhanced protein aggregation labeled in thioflavin T and Ubc9-RNAi exacerbated PFF-induced protein aggregates containing α-syn (Fig. 3*B*). Although high levels of α-syn in NC1 control were consistently detected in the thioflavin T-positive protein aggregates after PFF exposure, this trend was not shown to be statistically significant (Fig. 3*C*). Ubc9 overexpression enhanced the level of SUMOylation on α-syn, and SUMOylated α-syn was refractive to residual protein degradation without affecting transcriptional up-regulation, whereas Ubc9-RNAi reduced the protein level of α-syn (Fig. 5). Taken together, our results strongly support the report by Krumova et al. (2011) that SUMOylated α-syn promotes its solubility, prevents protein aggregation and further, reduces cytotoxicity in the SNpc. Our findings also support the recent publication that METH exposure reduces the level of SUMOylation on α-syn, and METH-induced α-syn aggregation is relieved by Ubc9 overexpression (Zhu et al., 2018). The study has also demonstrated that mutations in SUMOylation acceptor sites in α-syn enhance α-syn overexpression and aggregation induced by METH, which is mediated by impaired degradation through the UPS and the ALP *in vitro* and *in vivo* (Zhu et al., 2018). As our results suggest that diminished levels of SUMOylation are more prone to aggravate PFF-induced protein aggregation, it is in line with the recent DAT study (Cartier et al., 2019).

As shown in Figure 1, cell viability of Ubc9-overexpressing cells was significantly higher and its cytotoxicity was significantly lower than those of EGFP only cells after various concentrations of MPP+ or PFF exposure for 24 h. Although the mechanisms of suppressing ROS generation by pan-SUMOylation need to be characterized, there are numerous reports showing that SUMOylation plays a role in reducing oxidative stress. For example, SUMOylation may be an important regulatory mechanism that indirectly represses the production of ROS to ameliorate cellular stress (Pandey et al., 2011) and protects against oxidative stress with attenuation of stress-induced ROS generation by NADPH oxidase 2 (NOX2) inhibition (Kim et al., 2011a). Similarly, both conditional ablation and overexpression of Ubc9 induce functional impairment in mouse pancreatic β cells via interrupting ROS detoxification derived from NRF2 activity (He et al., 2018). Furthermore, the chemical inhibition of Ubc9 by 2-D08 induces ROS accumulation, which stimulates apoptosis in acute myeloid leukemia cells, probably via NOX2 deSUMOylation (Zhou et al., 2019). In addition, SUMO1 plays a critical role in regulating proper mitochondrial dynamics by protecting DRP1 protein which is required for mitochondrial fission (Harder et al., 2004). Hayashi et al. (2002) showed that the apoptotic process was triggered when SUMO1 conjugation was impaired. Therefore, there is mounting evidence to support that SUMOylation plays a critical role in regulating detoxification from oxidative stress to prevent cell death.

Previous *in vivo* studies have shown that animals with higher SUMO conjugation levels are more resistant to ischemic insult (Lee et al., 2011, 2014). In their study, Lee et al., modeled several lines of transgenic mice whose Ubc9 expression was broadly elevated to various degrees (Lee et al., 2011). These transgenic mice were observably more resistant to permanent middle cerebral artery occlusion (pMCAO), an animal stroke model, than corresponding WT animals. Higher Ubc9 levels in the brain resulted in lower infarction volumes under pMCAO (Lee et al., 2011). An *in vitro* study demonstrated that SUMOylation of a small portion of α-syn is sufficient to suppress its aggregation (Abeywardana and Pratt, 2015), which supports the previous study that a deficiency in the SUMOylation of α-syn augments its aggregation and thereby increases deleterious cellular toxicity (Krumova et al., 2011). However, other studies revealed the opposite conclusion that SUMOylation facilitates α-syn aggregation by blocking its ubiquitin-dependent degradation pathway and promoting its accumulation (Rott et al., 2017). Additionally, SUMO labeling of Lewy bodies in tissues from patients with PD and DLB was reported by Kim et al. (2011b), suggesting that SUMO was recruited to α-syn inclusions induced by proteasome inhibition. To resolve the discrepancy, we tested the effects of SUMOylaton on dopaminergic neurons against MPTP using pan-Ubc9-overexpressing Tg mice (Lee et al., 2011). In Ubc9-Tg mice, the deleterious effects by oxidative stress were strikingly reduced, as indicated that TH+ cell number in the SN and their projections to the striatum were significantly higher than those in WT mice, similarly treated with MPTP. As a follow-up, we also measured the levels of ROS from brain tissues to confirm our own *in vitro* results (Fig. 2); however, we could not detect meaningful ROS levels because of the lack of freshness of brain tissues. Because of quick redox reactions, we had technical difficulties in collecting reasonable levels of ROS from brain samples.

Our study demonstrates that Ubc9 overexpression increased the levels of SUMO1 and ubiquitin on α-syn (Fig. 9) and prevented MPP+ (MPTP)-induced SUMO1 removal from α-syn (*in vivo* data not shown); however, Ubc9 overexpression was not sufficient to block ubiquitin removal from α-syn (Fig. 9). In the previous DAT study, we have suggested that SUMO1 and ubiquitin may compete with each other to bind to lysine residues on DAT, although this hypothesis may be applied to certain proteins specifically (Cartier et al., 2019). More relevant to PD pathology, SUMOylation and ubiquitination may regulate α-syn degradation and pathologic aggregation reciprocally, suggesting its detrimental regulation by the competition (Rott et al., 2017). However, in our study, Ubc9 overexpression-induced SUMOylation may not counteract the ubiquitin binding to α-syn since the ubiquitin level of α-syn was upregulated by Ubc9 overexpression, not downregulated by the potential competition. Thus, the competition between SUMO and ubiquitin for binding to lysine residues in α-syn may be varied to regulate proteostasis, depending on the experimental conditions. In addition, the potential cross talk between deSUMO enzymes, sentrin-specific proteases (SENPs), and deubiquitinating enzymes (Ub-proteases) can be explored to understand the possible indirect competition (Liebelt and Vertegaal, 2016). Furthermore, the interplay between SUMOylation and phosphorylation in α-syn can be another interesting competition for preventing protein aggregation, although their targets are different amino acids (Shahpasandzadeh et al., 2014). Intriguingly, our results still support that SUMOylation delays the degradation of α-syn, yet it is not a pathologic process but rather prevents pathologic aggregation because of its high solubility (Krumova et al., 2011; Gupta et al., 2014).

In the previous study, we found that Ubc9 overexpression is independent of proteasomal degradation, yet it effectively prevents the PMA-mediated lysosomal degradation of DAT. The enhanced levels of DAT in the plasma membrane contribute to high functional activity of DAT in dopamine uptake (Cartier et al., 2019). Moreover, in our current α-syn study using the Chx-treated chase analysis, we verified that Ubc9 overexpression almost completely prevented the degradation of α-syn. The aberrant degradation was more likely mediated by the suppression of PKC-mediated lysosomal degradation than by the inhibition of proteasomal degradation, since the effect of PMA was annulled by Ubc9 overexpression, while the lack of MG132 effect was detected (Figs. 7,8; Sun et al., 2014). However, the inhibition of potential proteasomal degradation by SUMOylation cannot be excluded because MG132 may not be able to contribute to the additional inhibition because of the saturated level of Ubc9 effect. Therefore, we do not exclude the possible interruption by Ubc9 overexpression for proteasomal degradation of α-syn. Although Ubc9-mediated prevention of protein degradation may not occur exclusively through the lysosomal pathway and may be regulated by protein specificity, the inhibition of lysosomal degradation by SUMOylation was consistently identified in our DAT and α-syn studies. As a follow-up to better understand the idiopathic mechanisms of PD pathology, we are currently characterizing how SUMOs are removed from α-syn by PFF toxicity and determining which isoform of deSUMO enzymes, SENPs, is involved in detaching SUMOs from α-syn in the scope of understanding the idiopathic mechanisms of PD pathology (Liebelt and Vertegaal, 2016). These studies may reveal SUMOs or SENPs as novel regulatory targets that increase the protein solubility and prevent the formation of Lewy bodies in PD pathology.

## Synthesis

Reviewing Editor: Julie Andersen, Buck Institute Decisions are customarily a result of the Reviewing Editor and the peer reviewers coming together and discussing their recommendations until a consensus is reached. When revisions are invited, a fact-based synthesis statement explaining their decision and outlining what is needed to prepare a revision will be listed below. The following reviewer(s) agreed to reveal their identity: Han Seok Ko, Lori Buhlman.

Aggregation of alpha-synuclein is a hallmark pathology of Parkinson’s disease. The authors show data supporting that SUMOylation of alpha-synuclein maintains levels of soluble alpha-synuclein and prevent its aggregation. They show that mice overexpressing SUMO-conjugase Ubc9 are protected from MPTP exposure and provide evidence that Ubc9 expression reduces markers of cellular stress. This suggests that Ubc9- mediated SUMOylation could be an important mechanism in the regulation of alpha-synuclein solubility and degradation and a new therapeutic target of PD. The is novel and of general interest to the research community. The work is an interesting, timely, and well-executed study. On a technical note, extended data are felt to be integral to the study.

Specific comments:

Reviewer #1:

There are various grammatical mistakes that make some descriptions unclear all throughout the paper, so further grammatical proofreading is recommended. Some grammatical issues induce confusion especially when using language to describe the relative comparison of data between two groups’ results in the results section, as this is crucial to the reader’s understanding. This reviewer believe that the findings were very comprehensive and could be made even stronger if more additional in vivo experiments were done to evaluate Ubc9’s effects. Additionally, the significance statement can be improved to have a more clearly identifiable hypothesis as in its present form it seems more like just a summary of the study.

Specific points:

Figure 1.

1. Rather than redundantly restate the exact cell viability percentages shown in Fig. 1a again in writing, it is perhaps more useful to briefly state qualitatively what was seen instead and/or briefly relate this back to the hypothesis/mechanism suggested above. The same can be said for the description of data from Fig. 1b. For example, it could be mentioned that the 1280 μM concentration showed no difference in protective effects.

2. Please add N27 cells somewhere on the graph itself for Fig. 1c-f.

3. The authors should add WB data to show Ubc9 overexpression and Ubc9 knockdown in N27 cells.

4. The authors should check the effect Ubc9-RNAi on MPP+ induced cellular toxicity as well.

5. Throughout all the figures, please change Ubc9---> Ubc9 OE (overexpression).

Figure 2.

1. The authors should check the effect Ubc9-RNAi on MPP+ induced ROS levels.

Figure 3.

1. Immunostaining analysis with pS129 antibody and WB analysis with pS129 antibody using Triton X-100 soluble and insoluble fraction should be conducted as complementary analyses.

2. The authors should check the effect of UBC9 OE on PFF induced alpha-synuclein aggregation.

3. Based on Fig 5. several literatures suggest that endogenous alpha-synuclein requires PFF induced toxicity and alpha-synuclein aggregation formation and increased alpha-syn incubated with PFF exacerbates PFF induced neuronal toxicity and alpha-synuclein aggregation formation. As such it expects that the increase of alpha-synuclein protein due to Ubc 9 OE could exacerbate PFF induced neuronal toxicity and alpha-synuclein aggregation formation in N27 cells or the decrease of alpha-synuclein protein due to Ubc 9 RNAi could reduce PFF induced neuronal toxicity and alpha-synuclein aggregation formation in N27 cells. However, there are opposite results. Please the authors explain this inconsistency.

4. It seems that the aggregation test with Ubc9 overexpression is not included in the manuscript, but the abstract is marked as if it was done. Only Ubc9 RNAi knock down induced aggregation was shown. (From abstract, ‘In the mechanism of protection, Ubc9 overexpression significantly suppresses the toxicity-induced ROS generation and protein aggregation in Thioflavin T staining, compared to EGFP overexpression, whereas Ubc9-RNAi enhances the vulnerability.’). Please collect this.

Figure 4.

1. TH immunoreactivity in the STR n=8 is incorrect, should be n=7 in Figure legend. Please collect this.

2. Please change ST-> STR

3. The authors should add WB data to show Ubc9 overexpression in vivo.

4. Behavior should be conducted.

5. The knockdown effect of Ubc9 on MPTP induced toxicity should be monitored

6. The number of mouse TH neurons should be 6000∼7000 in one hemisphere.

7. TH DAB staining analysis should be instructive.

8. Intrastriatal PFF injection PD mouse model could be more instructive to claim the significance of Ubc9 on alpha-synuclein aggregation and DA degeneration due to SUMOylation.

Figure 5.

1. Representative images on Ubc9-RNAi should be added.

Reviewr #2:

• The manuscript needs to be carefully proofread for grammar and clarity. There are several instances where communication is unclear or improper grammar is used. For example, a sentence in the abstract reads, “Ubc9-mediated alpha-synuclein SUMOylation interrupts PMA-induced lysosomal degradation, which is protective due to its high solubility.” The sentence indicates that interruption of PMA-induced lysosomal degradation is protective due to high solubility of proteasomal degradation. An example of improper subject-verb agreement occurs in the second paragraph of the introduction: “Interestingly, UBC9 overexpression was reported to increase the solubility of α-synuclein and prevents Methamphetamine induced protein aggregation, and further non-SUMO α-synuclein mutants aggravate its aggregation by impairing proteasomal and lysosomal degradation (Zhu et al., 2018). “Methamphetamine” should not be capitalized, and it appears that there should be a comma after “further.” Verb tenses are mixed in the “Immunoprecipitation (IP)” description in the methods section. The intent of the last few sentences of the, “Ubc9 inhibition by RNAi enhances PFF-induced protein aggregation in Thioflavin T staining,” section needs to clarified.

• Define (spell out) PMA.

• Define SUMO

• “UBC9” is written in the second paragraph of the intro. Change it to “Ubc9,” to be consistent throughout the text.

• Describe the image analysis protocol for measuring CellROX fluorescence, thioflavin T, alpha-synuclein and tyrosine hydroxylase fluorescence. Include what represents “N.”

• Total protein stains are the preferred method to control western blot loading (https://www.ncbi.nlm.nih.gov/pubmed/?term=eaton+western+blot+loading). Using housekeeping genes and enzymatic signal amplification can distort quantification results.

• Two-way ANOVA should be run to test for statistical significance for data in figures 1 - 4 and 7 - 9 as there are two independent variables and multiple comparisons, for which the authors have not accounted.

• Means and SEMs are reported in the results section for some experiments and not others. I recommend removing them from the results section and including means and SEMs in figure captions for all data.

• In figure 2A, the thioflavin-positive staining in the right panel of the second row appears to occupy the alpha-synuclein positive parts of the cell. However, in the lower right “merged” panel, it appears that some of the alpha-synuclein staining is outside of the thioflavin-stained area. Please address this.

• The important comparison in figure 3 C is WGA/Glu + PFF in NC1 and Ubc-RNAi conditions, and it appears that the authors have acknowledged this at the end of the “Ubc9 inhibition by RNAi enhances PFF-induced protein aggregation...” section; however, this acknowledgement is unclear and should be rewritten.

• Authors should add a representative image of a western blot for data represented in 5C and D.

• Graphs in figures 1 - 5 and 9 are comprised of histograms with individual values, and graphs in figures 7 - 8 are floating boxes. Authors should represent data in a consistent format throughout the manuscript unless there is a need to do otherwise. A description of what is represented by the floating boxes should be added (i.e., do the bars represent data range, and boxes represent 95% CI?)

• Unlike with alpha synuclein IP followed by ubiquitin labeling, it looks like multiple forms of postranslationally modified alpha synuclein may have been captured in the blot in 9C. Address this.

Major

• Author should include evidence that the overexpression and knockdown of Ubc9 was achieved (e.g., western blotting with protein quantification).

• Authors should speculate on how Ubc9 overexpression decreases ROS.

• PMA is a PKC-activator that promotes lysosomal degradation. However, it is not clear whether PMA affects chaperone-mediated autophagy, macroautophagy, or both. Authors should more clearly address whether PMA is promoting chaperone-mediated autophagy or macroautophagy of alpha-synuclein. This information is critical in light of the authors’ conclusion that Ubc9-mediated SUMOlyation makes alpha-synuclein more soluble, and therefore less toxic, since chaperone-mediated autophagy is involved in soluble protein turnover, and macroautophagy is generally involved in the turnover of insoluble proteins (aggregates). Authors can genetically or pharmacologically block macroautophagy by knocking down autophagy genes or by applying bafilomycin, respectively. Knock-down of LAMP2A could allow observation of chaperone-mediated autophagy turnover of alpha-synuclein.

• Figure 9H shows that alpha synuclein ubiquitination increases with Ubc9 overexpression. Ubiquitin is typically conjugated to substrates for turnover. The authors should comment on why increased SUMOylation causes increase ubiquitination of alpha-synuclein, which does not appear to result in alpha-synuclein turnover.

• In the discussion, the authors indicate that they have data supporting that Ubc9 overexpression prevents MPP+-induced SUMO1 removal in vivo. These data would fit nicely in this paper.

Figure 6.

1. Although the isolation of degradation data without synthesis data via the use of Chx, was a sufficient way to test this, an additional step of testing whether it truly was due to the SUMOylation itself could have strengthened the claim potentially. For example, having a third manipulation of using a SUMOylation inhibitor (TAK-981 or some other feasible molecule) co-treatment with Ubc9 overexpression may have shown valuable data indicating alpha-syn levels in between the blue and green lines on Fig. 6b.

2. Throughout all the WB results, it is unclear why alpha-synuclein proteins were migrated to 19kDa. Alpha-synuclein should be migrated to 15 kDa. Please clarify this.

3. In Fig 6, 7, 8, and 9, the effect of UBC9 RNAi should be determined and please presents data.

Figure 7.

1. Subtitle (SUMOylation protects alpha-syn from proteasomal degradation) is too strong. There is no direct evidence. Should be town downed.

2. The last sentence in the “Cell culture and treatment” section in the Methods section needs to be elaborated with concentrations and times and more information rather than simply mentioning the co-treatment.

Figure 9.

1. Should check the effect of Ubc9 OE on the PFF-induced SUMO1 removal and ubiquitin removal from alpha-synuclein.

2. The authors suggested that SUMOlyation and ubiquitination are both increased in Ubc9 overexpression system. But due to their competitive feature on target sites, it would be better to conduct these experiments with residue specific antibodies or using a-syn constructs mutated with SUMOylation sites or ubiquitination sites.

3. The authors suggest that MPP+ treatment resulted in decreased SUMOylation (total lysate as well as immunoprecipitated a-syn) and decreased SUMOylation exacerbate PFF-induced a-syn accumulation. However, it is unclear how SUMOylation is decreased by MPP+ or PFF treatment. To that this, the authors should be check Ubc9 protein or mRNA level is downregulated both PFF or MPTP-treated neuron and SNpc of PFF or MPTP-injected mice.

There are several instances where a 2-way ANOVA should be used and t-tests or 1-way ANOVA is used instead.
