# Supplementary figures and images for: The SUMO Conjugase Ubc9 Protects Dopaminergic Cells from Cytotoxicity and Enhances the Stability of α-Synuclein in Parkinson’s Disease Models
Source: eNeuro. 2020 Sep 22;7(5):ENEURO.0134-20.2020. doi: 10.1523/ENEURO.0134-20.2020 (PMC7519168; doi:10.1523/ENEURO.0134-20.2020)

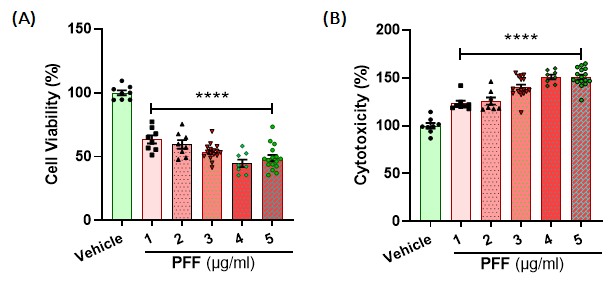

Supplement: Extended Data Figure 1-1 — The optimal toxic dose of α-syn PFF in N27 parental cells was assessed in the range of 1–5 μg/ml for cell viability (MTT; A) and cytotoxicity (LDH; B) assays. Our tests indicate that 1 μg/ml of PFF was consistently toxic to N27 cells, compared to vehicle-treated control (green bar) in both MTT and LDH assays. Scattered dot plots represent mean ± SEM (n = 8–16). One-way ANOVA, Dunnett’s test was applied for significance and vehicle was depicted for comparison; ****p < 0.0001. Download Figure 1-1, TIF file. [file enu-eN-NWR-0134-20-s01.tif]

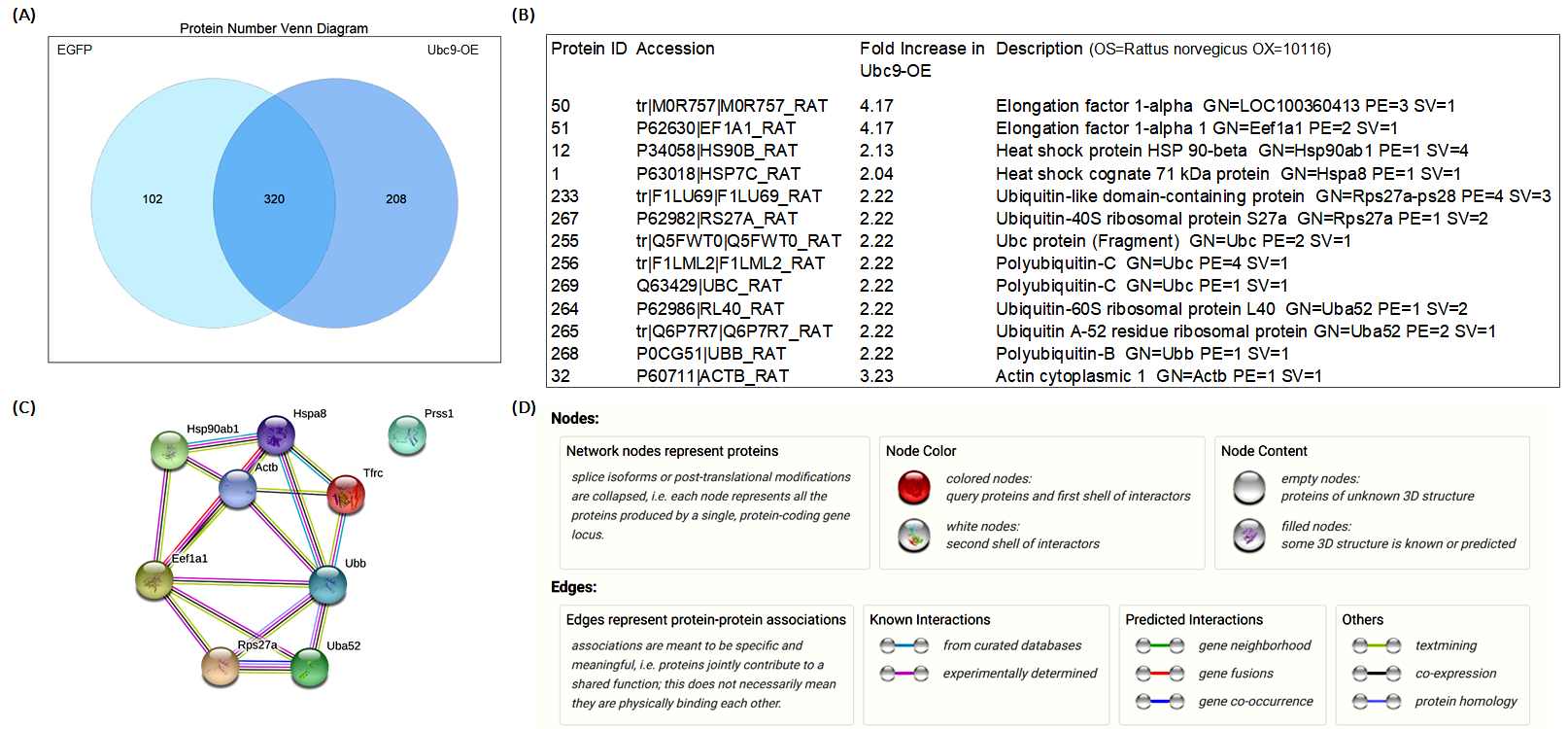

Supplement: Extended Data Figure 9-1 — Ubc9 overexpression increases several proteins’ interactions with α-syn in relative protein level analysis using mass spectrometry. A, The Venn diagram in the analysis of IPe’d α-syn from N27-EGFP and Ubc9-OE cell lysates shows that Ubc9-OE increases protein interaction with α-syn from 208 proteins, while Ubc9-OE decreases 102 proteins’ interactions with α-syn compared with EGFP cells. B, Several proteins in the list including ubiquitin were identified to interact with α-syn >2-fold higher in Ubc9 cells than in EGFP cells. C, A potential protein interaction schematic suggests that α-syn interacts with ubiquitin (Ubb and Uba52) 2.2-fold higher in Ubc9-OE than in EGFP only. D, Some protein interactions are considered as “known” and others are “predicted,” based on potential protein-protein interaction database (UniProt). Download Figure 9-1, TIF file. [file enu-eN-NWR-0134-20-s02.tif]
